# Comparison of MAPIE versus MAP in patients with a poor response to preoperative chemotherapy for newly diagnosed high-grade osteosarcoma (EURAMOS-1): an open-label, international, randomised controlled trial

**DOI:** 10.1016/S1470-2045(16)30214-5

**Published:** 2016-10

**Authors:** Neyssa M Marina, Sigbjørn Smeland, Stefan S Bielack, Mark Bernstein, Gordana Jovic, Mark D Krailo, Jane M Hook, Carola Arndt, Henk van den Berg, Bernadette Brennan, Bénédicte Brichard, Ken L B Brown, Trude Butterfass-Bahloul, Gabriele Calaminus, Heike E Daldrup-Link, Mikael Eriksson, Mark C Gebhardt, Hans Gelderblom, Joachim Gerss, Robert Goldsby, Allen Goorin, Richard Gorlick, Holcombe E Grier, Juliet P Hale, Kirsten Sundby Hall, Jendrik Hardes, Douglas S Hawkins, Knut Helmke, Pancras C W Hogendoorn, Michael S Isakoff, Katherine A Janeway, Heribert Jürgens, Leo Kager, Thomas Kühne, Ching C Lau, Patrick J Leavey, Stephen L Lessnick, Leo Mascarenhas, Paul A Meyers, Hubert Mottl, Michaela Nathrath, Zsuzsanna Papai, R Lor Randall, Peter Reichardt, Marleen Renard, Akmal Ahmed Safwat, Cindy L Schwartz, Michael C G Stevens, Sandra J Strauss, Lisa Teot, Mathias Werner, Matthew R Sydes, Jeremy S Whelan

**Affiliations:** a Stanford University School of Medicine and Lucile Packard Children's Hospital, Palo Alto, CA, USA; b Oslo University Hospital, Division of Cancer Medicine and Scandinavian Sarcoma Group, University of Oslo, Norway; c Institute for Clinical Medicine, University of Oslo, Norway; d Klinikum Stuttgart—Olgahospital, Cooperative Osteosarcoma Study Group (COSS), Stuttgart, Germany; e IWK Health Center, Dalhousie University, Halifax, NS, Canada; f Medical Research Council Clinical Trials Unit at University College London, London, UK; g Department of Preventive Medicine, Keck Medical Canter at the University of Southern California, Los Angeles CA, USA; h Children's Oncology Group, Arcadia, CA, USA; i Clinical Trials Research Unit, Institute of Clinical Trials Research, University of Leeds, Leeds, UK; j St James' Institute of Oncology, Leeds, UK; k Division of Pediatric and Adolescent Medicine, Mayo Clinic, Rochester, MN, USA; l Emma Children Hospital/Academic Medical Centre, Amsterdam, Netherlands; m Royal Manchester Children's Hospital, Manchester, UK; n Department of Pediatric Hematology/Oncology, Cliniques Universitaires Saint-Luc, Université Catholique de Louvain, Brussels, Belgium; o Department of Orthopaedics, University of British Columbia, Vancouver, BC, Canada; p Centre for Clinical Trials, University Hospital Muenster, Muenster, Germany; q Pädiatrische Hämatologie und Onkologie, Universitätsklinikum Bonn, Bonn, Germany; r Department of Radiology, Stanford University and Lucile Packard Children's Hospital, Palo Alto, CA, USA; s Skane University Hospital and Lund University, Lund, Sweden; t Department of Surgery, Dana-Farber Cancer Institute, Boston, MA, USA; u Dana-Farber/Boston Children's Cancer and Blood Disorders Center, Dana-Farber Cancer Institute, Boston, MA, USA; v Leiden University Medical Center, Leiden, Netherlands; w Institute of Biostatistics and Clinical Research, University of Muenster, Muenster, Germany; x Klinik für Allgemeine Orthopädie und Tumororthopädie, University of Muenster, Muenster, Germany; y Pädiatrische Hämatologie und Onkologie, University of Muenster, Muenster, Germany; z UCSF Medical Center-Mission Bay, Pediatric Oncology, San Francisco, CA, USA; aa Division of Pediatric Hematology-Oncology, The Children's Hospital at Montefiore, Bronx, NY, USA; ab Newcastle upon Tyne Hospitals NHS Trust, Newcastle upon Tyne, UK; ac Department of Oncology, Oslo University Hospital, Norwegian Radium Hospital, Scandinavian Sarcoma Group, Oslo, Norway; ad Seattle Children's Hospital, Fred Hutchinson Cancer Research Center, University of Washington, Seattle, WA, USA; ae Abt Pädiatrische Radiologie, AKK Altonaer Kinderkrankenhaus, Hamburg, Germany; af Center for Cancer and Blood Disorders, Connecticut Children's Medical Center, Hartford, CT, USA; ag Department of Pediatrics, St Anna Children's Hospital, Medical University Vienna, Vienna, Austria; ah University Children's Hospital Basel, Basel, Switzerland; ai Texas Children's Cancer Center, Baylor College of Medicine, Houston, TX, USA; aj Department of Pediatrics, UT Southwestern and Children's Medical Center, Dallas, TX, USA; ak Center for Childhood Cancer and Blood Disorders, Nationwide Children's Hospital and The Ohio State University, Columbus, OH, USA; al Division of Hematology, Oncology, and Blood and Marrow Transplantation, Department of Pediatrics, Children's Hospital Los Angeles, Los Angeles, CA, USA; am Keck School of Medicine, University of Southern California, Los Angeles, CA, USA; an Department of Pediatrics, Memorial Sloan Kettering Cancer Center, New York, NY, USA; ao University Hospital Motol, Pediatric Hematology/Oncology, Prague, Czech Republic; ap Clinical Cooperation Group Osteosarcoma, Pediatric Oncology Center, Department of Pediatrics, Technical University Munich, Munich, Germany; aq Department of Pediatric Oncology, Klinikum Kassel, Kassel, Germany; ar National Medical Center, Oncology Department, Budapest, Hungary; as Primary Childrens Hospital, The University of Utah, Salt Lake City, UT, USA; at HELIOS Klinikum Berlin-Buch, Klinik für Interdisziplinäre Onkologie, Berlin, Germany; au Department of Pediatric Hemato-oncology, University Hospital Leuven, Leuven, Belgium; av Aarhus University Hospital, Aarhus C, Denmark; aw Division of Pediatrics, The University of Texas M D Anderson Cancer Center, Houston, TX, USA; ax Bristol Royal Hospital for Children, Bristol, UK; ay Department of Oncology, University College Hospital, London, UK; az Department of Pathology, Boston Children's Hospital, Boston, MA, USA; ba HELIOS Klinikum Emil von Behring GmbH, Orthopädische Pathologie, Berlin, Germany

## Abstract

**Background:**

We designed the EURAMOS-1 trial to investigate whether intensified postoperative chemotherapy for patients whose tumour showed a poor response to preoperative chemotherapy (≥10% viable tumour) improved event-free survival in patients with high-grade osteosarcoma.

**Methods:**

EURAMOS-1 was an open-label, international, phase 3 randomised, controlled trial. Consenting patients with newly diagnosed, resectable, high-grade osteosarcoma aged 40 years or younger were eligible for randomisation. Patients were randomly assigned (1:1) to receive either postoperative cisplatin, doxorubicin, and methotrexate (MAP) or MAP plus ifosfamide and etoposide (MAPIE) using concealed permuted blocks with three stratification factors: trial group; location of tumour (proximal femur or proximal humerus *vs* other limb *vs* axial skeleton); and presence of metastases (no *vs* yes or possible). The MAP regimen consisted of cisplatin 120 mg/m^2^, doxorubicin 37·5 mg/m^2^ per day on days 1 and 2 (on weeks 1 and 6) followed 3 weeks later by high-dose methotrexate 12 g/m^2^ over 4 h. The MAPIE regimen consisted of MAP as a base regimen, with the addition of high-dose ifosfamide (14 g/m^2^) at 2·8 g/m^2^ per day with equidose mesna uroprotection, followed by etoposide 100 mg/m^2^ per day over 1 h on days 1–5. The primary outcome measure was event-free survival measured in the intention-to-treat population. This trial is registered with ClinicalTrials.gov, number NCT00134030.

**Findings:**

Between April 14, 2005, and June 30, 2011, 2260 patients were registered from 325 sites in 17 countries. 618 patients with poor response were randomly assigned; 310 to receive MAP and 308 to receive MAPIE. Median follow-up was 62·1 months (IQR 46·6–76·6); 62·3 months (IQR 46·9–77·1) for the MAP group and 61·1 months (IQR 46·5–75·3) for the MAPIE group. 307 event-free survival events were reported (153 in the MAP group *vs* 154 in the MAPIE group). 193 deaths were reported (101 in the MAP group *vs* 92 in the MAPIE group). Event-free survival did not differ between treatment groups (hazard ratio [HR] 0·98 [95% CI 0·78–1·23]); hazards were non-proportional (p=0·0003). The most common grade 3–4 adverse events were neutropenia (268 [89%] patients in MAP *vs* 268 [90%] in MAPIE), thrombocytopenia (231 [78% in MAP *vs* 248 [83%] in MAPIE), and febrile neutropenia without documented infection (149 [50%] in MAP *vs* 217 [73%] in MAPIE). MAPIE was associated with more frequent grade 4 non-haematological toxicity than MAP (35 [12%] of 301 in the MAP group *vs* 71 [24%] of 298 in the MAPIE group). Two patients died during postoperative therapy, one from infection (although their absolute neutrophil count was normal), which was definitely related to their MAP treatment (specifically doxorubicin and cisplatin), and one from left ventricular systolic dysfunction, which was probably related to MAPIE treatment (specifically doxorubicin). One suspected unexpected serious adverse reaction was reported in the MAP group: bone marrow infarction due to methotrexate.

**Interpretation:**

EURAMOS-1 results do not support the addition of ifosfamide and etoposide to postoperative chemotherapy in patients with poorly responding osteosarcoma because its administration was associated with increased toxicity without improving event-free survival. The results define standard of care for this population. New strategies are required to improve outcomes in this setting.

**Funding:**

UK Medical Research Council, National Cancer Institute, European Science Foundation, St Anna Kinderkrebsforschung, Fonds National de la Recherche Scientifique, Fonds voor Wetenschappelijk Onderzoek-Vlaanderen, Parents Organization, Danish Medical Research Council, Academy of Finland, Deutsche Forschungsgemeinschaft, Deutsche Krebshilfe, Federal Ministry of Education and Research, Semmelweis Foundation, ZonMw (Council for Medical Research), Research Council of Norway, Scandinavian Sarcoma Group, Swiss Paediatric Oncology Group, Cancer Research UK, National Institute for Health Research, University College London Hospitals, and Biomedical Research Centre.

## Introduction

Treatment strategies for high-grade osteosarcoma with multidrug chemotherapy and resection result in 3-year event-free survival of 60–70%.^1^, ^2^, ^3^, ^4^ The most common factors predicting survival are presence of metastases,^1^ histological response to preoperative chemotherapy,^1^, ^5^, ^6^, ^7^, ^8^ and complete surgical resection.^1^ Three of the active drugs in osteosarcoma^3^ include cisplatin,^9^, ^10^, ^11^ doxorubicin,^12^, ^13^ and high-dose methotrexate;^14^, ^15^ this combination (MAP), given preoperatively and postoperatively, is widely used for the treatment of osteosarcoma. Ifosfamide with^16^, ^17^ or without etoposide^17^ also has activity in this setting and when incorporated into the treatment of patients with metastatic disease seems to improve event-free survival.^18^ Though uncontrolled studies suggested that changing therapy on the basis of histological response improves outcomes,^3^, ^5^, ^19^ the efficacy of this strategy had not been tested in a randomised trial.

We report the primary results for patients who had a poor response and were randomised to the EURAMOS-1 trial, a collaboration between the Children's Oncology Group (COG), the Cooperative Osteosarcoma Study Group (COSS), the European Osteosarcoma Intergroup (EOI), and the Scandinavian Sarcoma Group (SSG). We did this trial to assess whether the addition of ifosfamide and etoposide to standard postoperative MAP would improve event-free survival in patients whose primary tumour showed a poor response to preoperative MAP.

**Research in context**

**Evidence before this study**

Osteosarcoma is a rare disease but is the most common bone tumour in children and adolescents and is associated with high mortality. Treatment for osteosarcoma involves multimodality therapy with chemotherapy followed by surgical resection and further chemotherapy. Before the EURAMOS-1 trial started, there has never been a study randomising patients with osteosarcoma following surgery to add chemotherapy to the preoperative backbone, and a formal literature review was not possible. Through collaborations and PubMed searches, we were aware of the relevant publications in osteosarcoma which were about importance of histological response and use of Ifosfamide in treating patients with a poor histological response. Histological response, as assessed by examination of the surgical specimen, is one of the strongest predictors of longer-term outcome for patients with osteosarcoma. Patients who have a poor response to chemotherapy (≥10% viable tumour) have a substantially worse survival than those with a good response (<10% viable tumour) with 5-year overall survival of around 45–55% and 75–80%, respectively. Several studies suggested that altering postoperative therapy might improve the outcome for patients with a poor histological response. Only one of the studies reporting improved outcome for patients with a poor response was randomised. However, the randomised study question was a comparison of upfront three-drug therapy versus three-drug therapy plus ifosfamide. The postoperative treatment for patients with a poor response was non-randomised and altered to include ifosfamide. Therefore, no previous study has assessed in a randomised comparison whether adding an ifosfamide containing combination to standard treatment improves the outcome for patients identified as having a poor histological response.

**Added value of this study**

Randomisation of patients with a poor response in EURAMOS-1 trial represents a large, international comparison, with patients registered at the start of treatment and randomly assigned after surgery. Preoperative and postoperative chemotherapy was with methotrexate, doxorubicin, and cisplatin (MAP), with half of the patients also assigned to receive ifosfamide and etoposide. Our findings do not support the intensification of postoperative chemotherapy by adding ifosfamide and etoposide for patients with a poor response to preoperative chemotherapy.

**Implications of all the available evidence**

Only a few randomised trials exist of this rare disease. The results of this randomised controlled trial will change clinical practice and help inform physicians' decisions when managing patients with osteosarcoma whose tumours show a poor histological response. Research is needed to improve outcomes for all patients with osteosarcoma and future trials need access to newer or targeted drugs, driven by improved understanding in the biology of the disease.

## Methods

### Study design and participants

EURAMOS-1 was an open-label, international, randomised, phase 3, controlled trial. The structure and design of this trial have been previously published.^20^ After a diagnostic biopsy, patients with newly diagnosed osteosarcoma could be registered to this trial. Patients were registered from 325 sites in 17 countries. The main eligibility criteria for registration included having high-grade localised or metastatic extremity or axial osteosarcoma deemed resectable by the treating team, age 40 years or younger at diagnostic biopsy; Karnofsky or Lansky status of at least 60, normal cardiac function (shortening fraction >28%), normal hearing, normal bone marrow as shown by an absolute neutrophil count of at least 1·5 × 10^9^ cells per L (or a white blood cell count of at least 3 × 10^9^ cells per L if neutrophil count is not available), and a platelet count of at least 100 000 platelets per μL. Patients were also required to have a serum bilirubin concentration of at most less than 1·5 times the upper limit of normal and a normal creatinine concentration for their age as per protocol. Enrolment criteria also included no previous treatment for osteosarcoma, and if the osteosarcoma was a second malignancy, patients could not have received previous chemotherapy. Patients' life expectancy was at least 3 months.

The main eligibility criteria for randomisation were registeration before definitive surgery to take part in EURAMOS-1; assessment of histological response in the primary tumour and randomisation within 35 days of definitive surgery (assessment by reference pathologist where possible); age 5 years or younger at biopsy for patients with good response; provision of all essential data (entry form, preoperative chemotherapy forms, surgery, and pathology report); receiving exactly two courses of cisplatin and doxorubicin, at least two courses, and no more than six courses of methotrexate; macroscopically complete surgical resection of the primary tumour; no evidence of local disease progression; recovery from previous therapy allowing administration of postoperative chemotherapy as planned; no progression of metastatic disease or new metastases and removal of metastases (in patients with metastatic disease at registration) done or deemed feasible (to be done after primary surgery); macroscopically complete surgical resection of the primary tumor; and written consent to participate in the study. The data was collected in each data centre (COG, COSS, EOI, and SSG) and transferred periodically to the coordinating data centre (Medical Research Council Clinical Trials Unit).

Before enrolment, all institutions were required to have obtained all regulatory and ethics approvals in accordance to their national, and for European centres, European rules and regulations, as mentioned in the protocol. All trial participants or legal guardians provided written informed consent before beginning protocol therapy. The protocol is available online.

### Randomisation and masking

Patients were randomly allocated (1:1) to receive either MAP or MAP plus ifosfamide and etoposide (MAPIE). Treatment allocation was done with centralised implementation of concealed random permuted blocks with three stratification factors: trial group, location of tumour (proximal femur or proximal humerus *vs* other limb *vs* axial skeleton), and presence of metastases (no *vs* yes or possible). Metastases were defined as at least three lesions bigger than 5 mm or a single lesion bigger than 1 cm. Patients with lung lesions not meeting these criteria were classified as having possible metastases. Patients were randomised centrally through Medical Research Council Clinical Trials Unit (COSS, EOI, SSG) and COG. The randomisation lists were prepared by the Medical Research Council Clinical Trials Unit for COSS, EOI, and SSG and by COG for COG sites. Each treating institution was responsible for enrolling their patients into the trial, and patients were assigned to interventions using the Medical Research Council Clinical Trials Unit randomisation system (for COSS, EOI, and SSG sites) and the COG randomisation system (for COG sites). Patients and investigators were not masked to treatment allocation.

### Procedures

Baseline assessment required a complete blood count, complete set of chemistry test data (including liver and kidney function), a baseline echocardiogram and hearing test, imaging with an MRI of the primary site, and a chest CT scan and a bone scan (a PET scan could be used instead). All patients received preoperative therapy with MAP^20^ (appendix p 10) for 10 weeks consisting of cisplatin 120 mg/m^2^ (4 h infusion of 60 mg/m^2^ per day for 2 days in COG sites; continuous 72 h intravenous infusion in other sites) and doxorubicin 37·5 mg/m^2^ per day on days 1 and 2 (on weeks 1 and 6). This was followed by high-dose methotrexate 12 g/m^2^ over 4 h (maximum dose 20 g at COG institutions) with hyper-hydration, alkalinisation, and standard leucovorin rescue at a dose of 15 mg/m^2^ starting 24–48 h from methotrexate infusion and continuing until methotrexate concentration was less than 0·1 μM (weeks 4, 5, 9, and 10).^20^, ^21^ Leucovorin rescue was adjusted with the dosing nomogram according to the methotrexate concentration. Patients were assessed with complete blood counts twice a week and with chemical tests (including liver and kidney function tests) before every cycle of chemotherapy. To begin myelosuppressive cycles, patients needed an absolute neutrophil count of at least 750 cells per μL and a platelet count of at least 75 000 platelets per μL. Criteria for administering high-dose methotrexate were different and included an absolute neutrophil count of at least 250 cells per μL and a platelet count of at least 50 000 platelets per μL.

Patients were restaged at 10 weeks preoperatively (with the same imaging studies done at diagnosis) and investigator-assessed response was reported according to Response to Treatment In Solid Tumors (RECIST) criteria, version 1. Those patients identified at their local institution to have no local or metastatic disease progression (defined as >20% increase in any primary tumour dimension associated with increased pain or inflammation) had primary tumour resection with centrally reviewed assessment of histological response.^22^, ^23^ Because all patients received the same preoperative therapy and treatment assignment was decided after pathology review, there was no masking. Eligibility for postoperative randomisation included macroscopic tumour resection, at least 10% morphologically viable tumour, no disease progression, no or resectable metastases, and ability to resume therapy within 35 days after surgery. We did not include central imaging review to confirm resectability of the disease or radiological response. We did not collect the percentage of viable tumour for each individual patient but rather whether they had at least 10% morphologically viable tumour.

Postoperative therapy is depicted in the appendix (p 10). Therapy consisted of MAP or MAPIE. Complete macroscopic resection of all disease for patients with initial metastases between weeks 11 and 20 was recommended. Imaging recommendations during treatment included a chest CT, a bone scan, and a plain radiograph of the primary tumour at 4-month intervals.

Cisplatin, doxorubicin, and methotrexate were administered at the same doses as given preoperatively. Ifosfamide (high dose 14 g/m^2^) at 2·8 g/m^2^ per day with equidose mesna uroprotection was administered, followed by etoposide 100 mg/m^2^ per day over 1 h on days 1–5 (three postoperative cycles). Two additional cycles of ifosfamide 3 g/m^2^ per day with mesna uroprotection on days 1–3 (9 g/m^2^) were given, with standard doxorubicin. Investigators were allowed to use supportive care with myeloid growth factor support according to local practice.

Patients treated with MAP received 29 weeks of treatment or four cycles of MAP and two cycles of methotrexate and doxorubicin (cisplatin was discontinued after a cumulative dose of 480 mg/m^2^). The protocol provided recommendations for dose reductions on the basis of the toxicity (from the common toxicity criteria) and therapy delays.

### Outcomes

The primary outcome measure was event-free survival, defined as the time from randomisation until first event (local recurrence, evidence of new or progressive metastatic disease, second malignancy, death, or a combination of those events) or censoring at last contact. Data were provided to the Medical Research Council by each group, but the outcome was not centrally reviewed. Secondary outcomes were overall survival (defined as the time from randomisation until death from any cause or last contact), short-term and long-term toxicity, and quality of life. Toxicity was measured by the treating institution and submitted to the Medical Research Council. Targeted toxicities included neutropenia, febrile neutropenia, fever without neutropenia, electrolyte abnormalities, cardiac dysfuction, renal dysfuction, and second malignancies. Serious adverse events were expedited (reported within 1 business day). Quality of life was assessed using the European Organization for Research and Treatment of Cancer quality of life questionnaire (EORTC QLQ-30; for patients at least 16 years of age) and the Pediatric Quality of Life inventory (PedsQL; for patients younger than 16 years of age). Questionaires were administered at baseline (before cycle 2), at week 20–22 (on active treatment), 18 months after start of treatment, and 3 years after starting treatment. Quality of life results will be published separately.

### Statistical analysis

With international accrual, we sought to randomise 693 patients with poor response to preoperative MAP. This and the partner trial in patients with a good response to preoperative MAP^19^, ^20^ were thought to require registration before chemotherapy of around 1400 patients. This number was increased to more than 2000 registered patients overall, after the observed randomisation rate was lower than anticipated.^19^ The study required at least 378 event-free survival events and at least 378 deaths to detect absolute improvements of 10% from 45% to 55% in 3-year event-free survival and 5-year overall survival (hazard ratio [HR] 0·75) with 5% two-sided significance levels and 80% power.^24^ The estimated sample size was calculated with the George and Desu method, and although this number of events was not reached, the independent data monitoring committee recommended early release of data because of the lower than predicted event rate and low likelihood that the planned number of events would be reached in a reasonable time. A preplanned subgroup analysis for patients with localised disease required 270 events to detect an 11% improvement, from 50% to 61% (HR 0·71) in 3-year event-free survival, and 5-year survival (two-sided significance level 5%, power 80%), assuming 85% of randomised patients would have localised disease (≥590 patients expected).

Primary and secondary outcomes were measured in the intention-to-treat population. Toxicity was assessed in the safety population which consisted of patients who started postoperative chemotherapy and who were assessed for toxicity. Statistical tests were done at a two-sided significance level of 0·05. The Kaplan-Meier method was used to estimate survival functions, log-rank test for differences between survival curves and Cox models (adjusted for stratification factors) to estimate the treatment effect. Median follow-up was calculated with reverse censoring on death. The χ^2^ test was used in a prespecified analysis to compare the proportion of patients with grade 3 or worse toxicity between the treatment groups. Interim analyses were done at regular intervals and presented to the independent data monitoring committee. The Haybittle-Peto stopping rule was used, and the trial was planned to stop if the p value for the event-free survival analysis was less than 0·001. The interim analyses were designed to examine safety of the patients, and therefore the protocol prespecified that if at interim analysis, the lower bound of the 95% CI for the proportion of patients in each group who died because of toxicity exceeded 3%, the future of the trial would be discussed with the Trial Steering Committee.

The proportionality of hazards for the treatment effect over time was tested. In the presence of non-proportionality, the difference between the groups was estimated with restricted mean survival time (RMST)^25^, ^26^ after fitting a flexible parametric model. RMST for event-free survival measures a mean time-to-first event, when time of consideration was restricted to t* years after randomisation; here t* was 6 years. The consistency of treatment effect in patients with localised or metastatic disease was tested by fitting a flexible parametric model with interaction between allocated treatment and metastases.

The treatment effect was estimated separately in the prespecified localised disease subgroup, and in the following subgroups defined post hoc: sex, age, site of disease, location of cancer on bone, and baseline metastases (lung and non-lung). Local recurrence, new metastases, progression of existing metastases, death, or a combination of those events were considered a competing event for reporting second malignancy. Analyses were done with Stata version 14.0. This trial is registered with ClinicalTrials.gov, number NCT00134030.

### Role of the funding source

The funders of this study had no role in study design, data collection, data analysis, data interpretation, or writing of the report. MRS and GJ had access to raw data. The corresponding author had full access to all data in the study and all authors had final responsibility for the decision to submit for publication.

## Results

**Figure 1:**
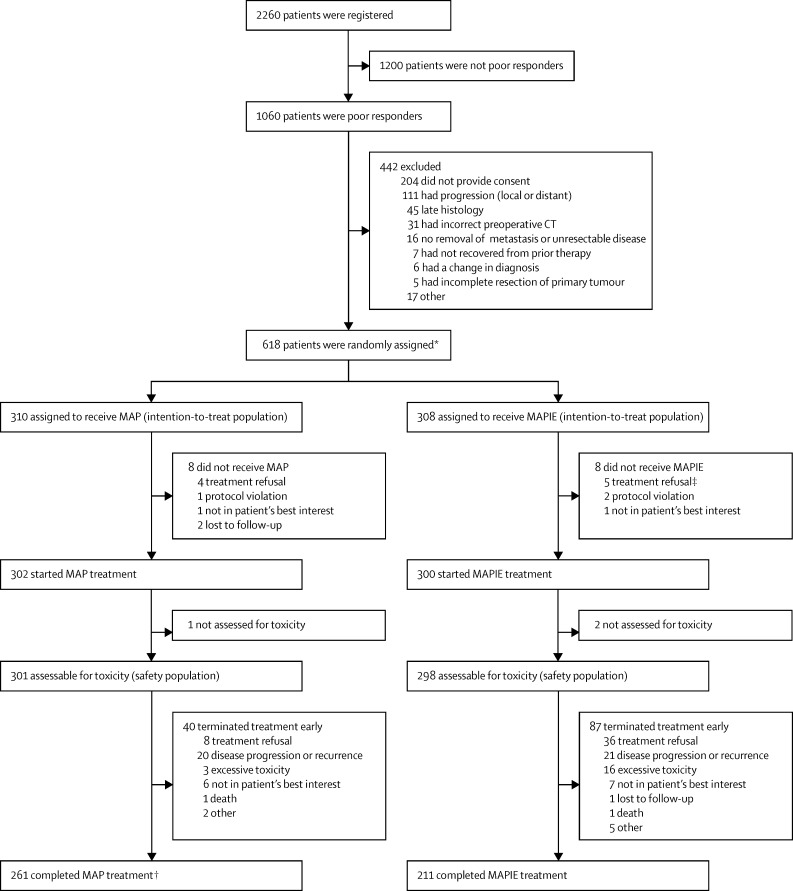
Trial profile *We later found out that one patient actually had a good histological response (allocated to MAP group at randomisation and analysed as in the MAP group). †One additional patient completed MAP (as he was not assessed for toxicity, he was excluded previously in this diagram). ‡One patient was lost to follow-up at randomisation.

Between April 14, 2005, and June 30, 2011, 2260 patients were registered until the overall recruitment target was reached. The dataset was frozen on Nov 19, 2014. 618 patients were randomly assigned from the poor responders group: 310 to receive MAP and 308 to receive MAPIE (figure 1); this was lower than the anticipated target of 693. The main reasons for non-randomisation were no provision of consent (appendix p 4). Baseline characteristics at registration for the randomly assigned patients were similar between the groups (table 1). Median follow-up for the entire trial population was 62·1 months (IQR 46·6–76·6); 62·3 months (IQR 46·9–77·1) for the MAP group and 61·1 months (IQR 46·5–75·3) for the MAPIE group. For patients last reported alive and not lost to follow-up, 325 (87%) of 373 had follow-up within 14 months before the data freeze. 29 (9%) of 310 patients in MAP and 23 (7%) of 308 patients in MAPIE were permanently lost to follow-up more than 14 months before the data freeze.

**Figure 2:**
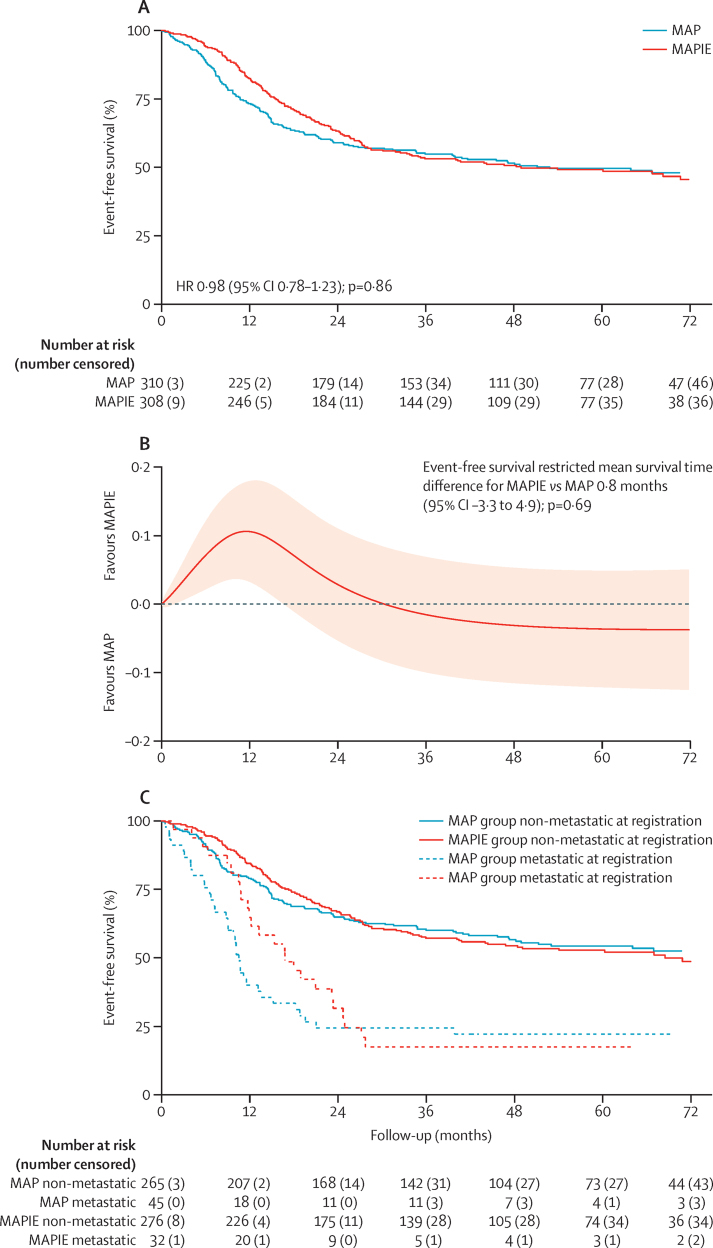
Event-free survival (A) Kaplan-Meier curve of event-free survival. (B) Absolute difference in event-free survival by flexible parametric model difference. 95% CI is shown by shading. (C) Kaplan-Meier curve of event-free survival by metastases status at registration.

307 event-free survival events were reported: 153 in the MAP group and 154 in the MAPIE group. The HR for event-free survival comparison in MAPIE versus MAP was 0·98 (95% CI 0·78–1·23, p=0·86), but the proportionality of hazards assumption did not hold (p=0·0003); therefore, we estimated event-free survival with the RMST approach. Mean time to first event was 43·3 months (95% CI 40·1–46·4) for patients allocated to the MAP group, and 44·1 months (41·1–47·1) for patients allocated to the MAPIE group, over a period of 6 years from randomisation. The difference in estimated RMST was 0·8 months (95% CI −3·3 to 4·9, p=0·69; figure 2). The 3-year event-free survival estimates were 55% (95% CI 49–60) in the MAP group and 53% (47–59) in the MAPIE group (figure 2). In both groups, the first event mostly involved metastases—133 (87%) of 153 patients in the MAP group and 129 (84%) of 154 patients in the MAPIE group.

247 event-free survival events were reported in 541 patients with localised disease at the time of registration (118 in the MAP group and 129 in the MAPIE group; figure 2). 3-year event-free survival estimates in localised disease were 60% (95% CI 54–66) in the MAP group and 57% (51–63) in the MAPIE group. The HR was 1·03 (0·81–1·33, p=0·80) but with considerable non-proportionality (p=0·0071). The difference in estimated RMST between the MAPIE and MAP groups was 0·05 months (−4·7 to 4·8; p=0·98; figure 2). 3-year event-free survival in patients who had metastases at registration was 24% (13–38) in the MAP group and 18% (6–33) in the MAPIE group. The event-free survival comparison in various subgroups is shown in the appendix (p 7). No evidence of heterogeneity of a treatment effect across the explored subgroups was observed.

**Figure 3:**
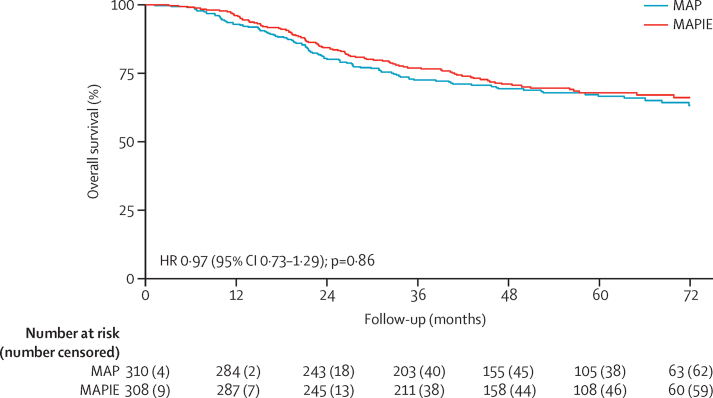
Kaplan-Meier curve of overall survival

193 deaths were reported (101 in the MAP group *vs* 92 in the MAPIE group). Survival data are immature. With current data, the HR estimate was 0·97 (95% CI 0·73–1·29, p=0·86) and at 3-years, overall survival was 72% (95% CI 67–77) for the MAP group and 77% (72–81) for the MAPIE group (figure 3).

Details of received standardised postoperative chemotherapy doses, the number of patients who received the target number of chemotherapy doses, and the number of patients who received at least 80% of the planned dose are shown in table 2. Chemotherapy compliance was generally poorer with MAPIE than with MAP. Of the 77 patients with metastases at registration, 75 of 77 either had resection or resection was no longer needed and two of 75 were not operated on (one in the MAPIE group due to clinician's and patient's choice group *vs* one in the MAP group who was inoperable).

Toxicity data are available for 301 (99·7%) of 302 MAP and 298 (99·3%) of 300 patients in the MAPIE group who started treatment (table 3), and the remaining three patients were not assessed for toxicity (one in the MAP group and two in the MAPIE group). Less commonly reported postoperative toxicities of grade 3 or 4, outside of those routinely solicited, are given in the appendix (p 8); information about raised liver enzymes, nausea, vomiting, and diarrhoea were not routinely collected, but were reported at the discretion of the site and are taken into consideration when highest grade toxicity reported was determined (table 4). The grades of the toxicities recorded during postoperative chemotherapy were similar between different treatment groups: worst toxicity of grade 3 or above was reported by 287 (95%) of 301 patients in MAP and 281 (94%) of 298 in MAPIE group (table 4). MAPIE was associated with more frequent grade 4 non-haematological toxicity (35 [12%] of patients in the MAP group *vs* 71 [24%] of 298 patients in the MAPIE group), mainly because of infections with absolute neutrophil count of less than 1 × 10^9^ neutrophils per L, febrile neutropenia without documented infection, and hypophosphataemia (table 4). Two patients died during postoperative therapy, one due to infection although they had a normal absolute neutrophil count (MAP group), and one due to left ventricular systolic dysfunction (MAPIE group). One suspected unexpected serious adverse reaction was reported in the MAP group: bone marrow infarction due to methotrexate. 19 patients discontinued because of drug-related toxicity; three in the MAP group and 16 in the MAPIE group. Dose was reduced or delayed according to criteria in the protocol for 176 (58%) of 301 patients in the MAP group and 184 (62%) of 298 patients in the MAPIE group. Three patients were not assessed for dose reduction or delay, one in the MAP group and two in the MAPIE group.

Left ventricular systolic dysfunction grade 3 events were reported in three (2%) of 134 patients in the MAPIE group and one (1%) of 136 in the MAP group. Long-term toxicity data was available from COSS, EOI, and SSG centres and reflects adverse events which might or might not be treatment related. Creatinine grade 3 events were reported for 3 (2%) out of 138 patients in the MAPIE group and no patients in MAP. Urinary electrolyte wasting grade 3 was reported for five (4%) of 134 patients in the MAPIE group and no patients in the MAP group. Renal failure grade 3 (four [3%] of 138 patients) and grade 4 (one [1%] of 138 patients) events were reported in the MAPIE group but not in the MAP group. There was no consistent pattern of reversibility for left ventricular systolic dysfunction or renal events, as some patients improved, whereas others did not, but the number of events was too small to analyse further.

13 second primary malignancies were reported (three in the MAP group *vs* ten in the MAPIE group; appendix pp 5, 6). Cause-specific HR estimate for MAPIE versus MAP was 3·24 (0·87–12·06), p=0·079; subdistribution HR was 3·21 (0·87–11·85), p=0·081; appendix p 6). Most of the second malignancies were myeloid (myelodysplasia and acute myeloid leukaemia): two of three in the MAP group and eight of ten in the MAPIE group, mostly recording cytogenetic abnormalities causally associated with administration of alkylating drugs (monosomy-7 or chromosome-5 abnormalities) or etoposide (11q23 abnormalities). One of three patients in the MAP group and two of ten patients in the MAPIE group had second solid tumours. A longer follow-up will allow for more precise estimates.

## Discussion

Standard treatment for high-grade osteosarcoma includes preoperative chemotherapy followed by surgical resection and postoperative chemotherapy.^1^, ^2^, ^3^, ^4^, ^8^ Although previous uncontrolled studies^3^, ^5^, ^19^ suggested that altering therapy on the basis of histological response improved outcome, this had not been tested in randomised controlled trials. EURAMOS-1 is, to the best of our knowledge, the first collaboration to assess in a randomised manner whether altering chemotherapy on the basis of histological response improves outcome for patients with osteosarcoma. Our results for patients with a poor histological response show that the addition of ifosfamide and etoposide to standard postoperative therapy does not improve outcome and rather increases the incidence of toxic effects.

A previous randomised controlled trial^29^ of around 240 patients assessed the addition of ifosfamide to standard MAP chemotherapy for patients with osteosarcoma. This trial included an upfront randomisation to MAP versus MAP plus ifosfamide, with postoperative ifosfamide given to those who had received it preoperatively or whose tumour showed a poor histological response to preoperative MAP (<90% necrosis). No significant difference in outcome was observed between the two groups and the timing of the addition of ifosfamide made no difference to outcome. On the basis of these data and our EURAMOS results, we recommend continuing with the same chemotherapy as used preoperatively for patients with a poor histological response to preoperative treatment.

Our results are also consistent with the results of a previous randomised controlled trial^2^ by the COG, which COG assessed the role of ifosfamide and mifamurtide in patients with osteosarcoma. The study assessed whether the addition of ifosfamide or mifamurtide to the MAP regimen improved long-term outcomes; patients were treated preoperatively with either MAP or MAP plus ifosfamide, and subsequently postoperatively treated with or without mifamurtide. No difference in histological response was observed after the preoperative period, and no difference in event-free survival or overall survival was observed between the two chemotherapy groups. Our results are also consistent with the conclusion of a meta-analysis^30^ where outcomes for osteosarcoma patients were improved with the use of at least three drugs, but no improvement was noted with the use of MAP plus ifosfamide versus MAP.

EURAMOS-1 was complicated by a lower than anticipated randomisation rate, probably related to the timing of the randomisation, close to the time of the intervention, after patients had been exposed to significant treatment-related toxicities. Non-randomisation was 42% in known poor responders and occurred in all four trial groups (COG, COSS, EOI, and SSG) ranging between 33% and 45%. Disease progression during preoperative treatment was reported in some patients, who were ineligible for randomisation. Four patients were considered ineligible at retrospective central pathology review. These patients were included because the treating clinician thought them eligible. This highlights the diagnostic difficulties facing physicians, pathologists, and patients.

As anticipated, a larger number of MAPIE-treated patients developed second malignancies than patients treated with MAP, although the difference was not statistically significant. Most of these second malignancies were myeloid in origin, which is not unexpected given the short follow-up and the natural history of therapy-related myeloid disorders.^31^, ^32^ The cytogenetic abnormalities in some of these leukaemias were consistent with exposure to alkylating drugs^32^ and etoposide.^33^ Although second solid malignancies are more common after combined modality therapy, including irradiation,^34^ alkylating drugs also contribute to this risk. Therefore, continued follow-up is crucial to identify any second solid malignancies occurring as later events in this patient population. Overall survival will additionally be analysed with longer follow-up.

The number of events was lower than anticipated, as event-free survival results were based on 307 event-free survival events, rather than the planned 378; event rate was lower than predicted and our 3-year event-free survival estimate for MAP was 55% (95% CI 49–60) rather than 45%. Follow-up visits started later in the MAPIE group because treatment was longer (40 weeks *vs* 29 weeks). Therefore early imaging assessments might not have been identically timed by group, resulting in the apparent early separation observed in the event-free survival curve in favour of the MAPIE group.

The timing of our intervention might have been too late because the first cycle of ifosfamide plus etoposide was administered at week 17 (5 weeks from resumption of postoperative therapy). Also, the overall intensity of therapy might have contributed to the inability of MAPIE-treated patients to receive all intended treatment. Ultimately, the best strategy for patients with a poor histological response might not be therapy intensification since this approach has been unsuccessful in childhood soft tissue sarcomas^35^ and in a previous study in osteosarcoma.^4^ The use of functional imaging early during treatment might identify patients who will have a poor response and allow incorporation of alternative treatment strategies earlier. This technique has been assessed in patients with osteosarcoma^36^ and found to predict both histological response and outcome.

We included patients with both initially localised and initially metastatic disease in the same trial because it was not always possible to differentiate up-front between patients with localised disease and those with metastatic disease, and we have therefore also included patients with “possible” metastatic disease; other prognostic factors are the same in patients with both metastatic and localised disease. The inclusion of patients with initial metastases into the EURAMOS-1 trial was unique. The patients with localised disease needed to have no metastatic progression during preoperative therapy to be eligible for randomisation. Additionally, these patients had to undergo resection of all metastatic lesions during the postoperative period. The 3-year event-free survival for patients with metastases was 24% (13–38) in the MAP group and 18% (6–33) in the MAPIE group, and these results are similar to those of other published series.^37^, ^38^ The EURAMOS-1 trial included only 77 patients with metastases, which makes it difficult to draw firm conclusions regarding the outcome for these patients. Additionally, since the EURAMOS-1 patients with metastases needed to have resectable disease, the population included might not be entirely comparable to those included in the previous trials.

Our trial has several strengths, including being an international randomised controlled trial in a rare disease with widely applicable results. It also has several limitations including the lower-than-expected acceptance of randomisation and the lower-than-predicted observed event rate. The percentage of viable tumours^22^, ^23^ at the individual patient level was not collected in this study. This limits our ability to assess overall prognosis by percentage of viable tumour. Because the study question was whether altering therapy on the basis of histological response improved outcome, we do not think collection of those data is crucial to the study results overall. Another potential limitation is the fact that we did not include central imaging review. This could have resulted in local sites removing patients from the study for apparent local disease progression, which might actually have had represented only pseudoprogression. In addition, the inclusion of patients deemed to have resectable metastases without undergoing central review increases the possibility that those patients did not truly have resectable disease. The fact that a higher proportion of patients with metastatic disease were not randomised raises the possibility that some local institutions misjudged the ability to do a complete resection. The number of patients removed from protocol therapy because metastases were unresectable might be anticipated to be balanced between the treatment groups. Lastly, although we collected total dose received by each patient, we did not collect it in a way that allows us to present relative dose intensity received. This is a potential limitation especially because patients treated in the experimental group appeared to receive less of the intended therapy.

The addition of mifamurtide to the MAP chemotherapy regimen for patients with localised osteosarcoma resulted in improved overall survival.^2^ The drug was approved for use in Europe on the basis of those results, but has not been approved for use in the USA. However, the results suggest that assessment of drugs such as mifamurtide that stimulate the immune system might improve outcome. We have subsequently reached an apparent plateau in outcomes for patients with osteosarcoma.^39^ The development of strategies to better understand the biology of this tumour^40^ might aid in the identification of drug targets. Collaboration with veterinary oncologists might speed up target identification in human beings as canine osteosarcoma is more common than human osteosarcoma.^41^ Genome-wide analysis of osteosarcoma samples might also help identify important targetable genetic mutations.^42^ The identification of genetic alterations that could be used as therapeutic targets is an important step to develop better treatment approaches.

Overall, EURAMOS-1 showed that treatment with MAPIE resulted in similar event-free survival to MAP, results in increased toxicity, decrease in total received doses, and more second malignancies. Therefore, we argue strongly against adding ifosfamide and etoposide to the backbone of MAP therapy for patients whose tumour shows a poor response to preoperative treatment. Future strategies might seek to incorporate drugs with different mechanisms of action either alongside postoperative chemotherapy or earlier in the treatment pathway of appropriately identified patients. Such trials will require continued international collaboration by the EURAMOS group.

## Figures and Tables

**Table 1:** Baseline characteristics

		**MAP (n=310)**	**MAPIE (n=308)**
Sex			
	Male	174 (56%)	191 (62%)
	Female	136 (44%)	117 (38%)
Age at registration (years)			
	<5	1 (<1%)	0
	5–9	54 (17%)	40 (13%)
	10–19	204 (66%)	231 (75%)
	20–29	35 (11%)	32 (10%)
	≥30	16 (5%)	5 (2%)
	Median (IQR)	15 (11–17)	14 (12–17)
	Range	4–40	5–39
Site of tumour			
	Femur	154 (50%)	166 (54%)
	Tibia	75 (24%)	76 (25%)
	Fibula	17 (5%)	13 (4%)
	Humerus	39 (13%)	27 (9%)
	Radius	4 (1%)	6 (2%)
	Ulna	2 (1%)	1 (<1%)
	Scapula/clavicle	3 (1%)	2 (1%)
	Pelvis/sacrum	8 (3%)	11 (4%)
	Rib	3 (1%)	3 (1%)
	Other	5 (2%)	3 (1%)
Location of tumour on the bone			
	Proximal	114 (37%)	109 (35%)
	Diaphysis	11 (4%)	12 (4%)
	Distal	166 (54%)	168 (55%)
	N/A (not long bone)	19 (6%)	19 (6%)
Pathological fracture at diagnosis			
	No	276 (89%)	270 (89%)
	Yes	34 (11%)	35 (11%)
	Data missing	0	3
Localised disease		265 (85%)	276 (90%)
Lung metastases			
	No*	272 (88%)	280 (91%)
	Yes	38 (12%)	28 (9%)
Extra-pulmonary metastases			
	No*	302 (97%)	299 (97%)
	Yes	8 (3%)	9 (3%)
Histological classification†			
	Conventional	288 (94%)	289 (95%)
	Telangiectatic	11 (4%)	6 (2%)
	Small cell	3 (1%)	2 (1%)
	High-grade surface	5 (2%)	6 (2%)
	Periosteal‡	0	1 (<1%)
	Biopsy data not available	3	4

MAP=methotrexate, doxorubicin, and cisplatin. MAPIE=MAP plus ifosfamide and etoposide. Missing values are not included in the percentage calculation.

* Possible metastases were combined with no metastases.

† Histological classification was based on diagnostic biopsy according to the WHO 2002 classification of osteosarcoma.^27^, ^28^

‡ Patient with periosteal classification was considered eligible at registration.

**Table 2:** Summary of patients who received postoperative MAP and MAPIE

	**Target cumulative standardised dose**	**Median cumulative standardised dose (IQR)**		**Target number of doses**	**Patients (%) who received target number of doses**		Received at least 80% of planned dose*	
		MAP	MAPIE		MAP (n=302)	MAPIE (n=300)	MAP (n=302)	MAPIE (n=299)†
Methotrexate (g/m^2^)	96	93·9 (80·0–97·1)	87·9 (67·7–96·6)	8–10	226 (75%)	178 (59%)	235 (78%)	194 (65%)
Doxorubicin (mg/m^2^)	300	296 (284–303)	299 (233–305)	4	250 (83%)	233 (78%)	245 (81%)	221 (74%)
Cisplatin (mg/m^2^)	240	239 (235–241)	240 (230–244)	2	277 (92%)	251/299 (84%)‡	267 (88%)	238/298 (80%)‡
Ifosfamide 14 g (g/m^2^)	42	NA	40·8 (26·6–42·4)	3	NA	222 (74%)	NA	204 (68%)
Ifosfamide 9 g (g/m^2^)	18	NA	17·4 (8·9–18·3)	2	NA	193 (64%)	NA	190 (64%)
Etoposide (g/m^2^)	1·5	NA	1·47 (1·00–1·52)	3	NA	224 (75%)	NA	211 (71%)

All patients received preoperative treatment. MAP=methotrexate, doxorubicin, and cisplatin. MAPIE=MAP plus ifosfamide and etoposide. NA=not applicable.

* Percentages were calculated by dividing by the number of patients who received at least one dose of study drug.

† Doses for MAPIE drugs are not known for one patient who started MAPIE treatment; number of received cycles for each drug is known.

‡ Received cisplatin dose and number of cycles are not known for one patient.

**Table 3:** Postoperative treatment-related adverse events

	**MAP (n=301)**				**MAPIE (n=298)**			
	Grade 1–2	Grade 3	Grade 4	Grade 5	Grade 1–2	Grade 3	Grade 4	Grade 5
Any toxicity	11 (4%)	26 (9%)	260 (86%)	1 (<1%)	12 (4%)	20 (7%)	260 (87%)	1 (<1%)
Non-haematological event*	57 (19%)	197 (65%)	35 (12%)	1 (<1%)	24 (8%)	187 (63%)	71 (24%)	1 (<1%)
Infection in patients with absolute neutrophil count ≥1 × 10^9^ neutrophils per L	35/209 (17%)	48/209(23%)	1/209 (<1%)	1/209 (<1%)	29/227 (13%)	57/227 (25%)	9/227 (4%)	0
Left ventricular systolic dysfunction	42/290 (14%)	2/290 (1%)	0	0	59/292 (20%)	3/292 (1%)	0	1/292 (<1%)
Neutropenia	16 (5%)	21 (7%)	247 (82%)	0	11/295 (4%)	16/295 (5%)	252/295 (85%)	0
Thrombocytopenia	44/298 (15%)	50/298 (17%)	181/298 (61%)	0	26/297 (9%)	36/297 (12%)	212/297 (71%)	0
Febrile neutropenia without documented infection	0/299	138/299 (46%)	11/299 (4%)	0	0	182/297 (61%)	35/297 (12%)	0
Anaemia	†	19 (6%)	11 (4%)	0	†	17 (6%)	17 (6%)	0
Documented infection in patients with absolute neutrophil count <1 × 10^9^ neutrophils per L	19/300 (6%)	104/300 (35%)	4/300 (1%)	0	10/297 (3%)	135/297 (45%)	23/297 (8%)	0
Leucopenia	†	2 (1%)	9 (3%)	0	†	2 (1%)	18 (6%)	0
Hypophosphataemia	70/286 (24%)	39/286 (14%)	4/286 (1%)	0	76/292 (26%)	59/292 (20%)	13/292 (4%)	0
Mucositis or stomatitis	118/267 (44%)	84/267 (31%)	6/267 (2%)	0	129/262 (49%)	70/262 (27%)	9/262 (3%)	0
Hypokalaemia	†	1 (<1%)	0	0	†	3 (1%)	7 (2%)	0
Mood alteration	70/297 (24%)	2/297 (1%)	3/297 (1%)	0	74/297 (25%)	10/297 (3%)	3/297 (1%)	0
Hypomagnesaemia	†	1 (<1%)	3 (1%)	0	†	0	1 (<1%)	0
Abnormal creatinine concentration	41/300 (14%)	2/300 (1%)	1/300 (<1%)	0	55/298 (18%)	5/298 (2%)	2/298 (1%)	0
Infection with unknown absolute neutrophil count	†	3 (1%)	1 (<1%)	0	†	4 (1%)	1 (<1%)	0
Electrolyte abnormalities	†	2 (1%)	1 (<1%)	0	†	4 (1%)	1 (<1%)	0
Infection with grade 3 or 4 absolute neutrophil count	†	3 (1%)	1 (<1%)	0	†	2 (1%)	1 (<1%)	0
Thrombosis, thrombus, or embolism	†	2 (1%)	1 (<1%)	0	†	1 (<1%)	1 (<1%)	0
Pain	†	10 (3%)	1 (<1%)	0	†	11 (4%)	0	0
Abnormal bilirubin concentration‡	53/147 (36%)	7/147 (5%)	0	0	63/147 (43%)	8/147 (5%)	1/147 (1%)	0
Encephalopathy	†	3 (1%)	0	0	†	3 (1%)	1 (<1%)	0
Hyperglycaemia	†	3 (1%)	0	0	†	2 (1%)	1 (<1%)	0
Metabolic or other laboratory-confirmed event	†	2 (1%)	0	0	†	3 (1%)	1 (<1%)	0
Seizure	4/300 (1%)	0	1/300 (<1%)	0	9/297 (3%)	1/297 (<1%)	0	0
Motor neuropathy	4/298 (1%)	12/298 (4%)	0	0	9/296 (3%)	11/296 (4%)	0	0
Hearing	60/270 (22%)	8/270 (3%)	0	0	74/274 (27%)	2/274 (1%)	0	0
Sensor neuropathy	39/299 (13%)	5/299 (2%)	0	0	31/297 (10%)	5/297 (2%)	0	0
Somnolence	2/300 (1%)	1/300 (<1%)	0	0	17/295 (6%)	6/295 (2%)	0	0
Confusion	5/298 (2%)	0	0	0	19/296 (6%)	6/296 (2%)	0	0
Typhlitis	6/298 (2%)	2/298 (1%)	0	0	8/292 (3%)	4/292 (1%)	0	0
Allergic reaction	†	2 (1%)	0	0	†	4 (1%)	0	0
Urinary electrolyte wasting	20/275 (7%)	0	0	0	22/266 (8%)	4/266 (2%)	0	0
Glomerular filtration rate	22/255 (9%)	0	0	0	36/263 (14%)	1/263 (<1%)	0	0
Haemorrhage, genitourinary bladder	13/297 (4%)	0	0	0	22/294 (7%)	1/294 (<1%)	0	0

All grade 3–5 adverse events are shown for all routinely collected toxicities; additionally, any toxicity of a type that was not routinely solicited on the case report forms, but was reported for at least five patients is included. Grade 1–2 adverse events are also included if reported for at least 10% of patients. Percentages for each toxicity were determined by dividing by the total number of patients who had information about that particular toxicity. Three patients (one receiving MAP and two receiving MAPIE) were not assessed for toxicity and are not included in the table above. MAP=methotrexate, doxorubicin, and cisplatin. MAPIE=MAP plus ifosfamide and etoposide.

* Any toxicity, excluding neutropenia, thrombocytopenia, anaemia, and leucopenia.

† Toxicities not routinely solicited; sites could only report these under “other” toxicities.

‡ Bilirubin data was collected by the Cooperative Osteosarcoma Study Group, the European Osteosarcoma Intergroup, and the Scandinavian Sarcoma Group sites only.

**Table 4:** Summary of worst grade toxicity during postoperative chemotherapy

	**MAP (n=301)**				**MAPIE (n=298)**				χ^2^ test p value*
	Grade 3	Grade 4	Grade 5	Total grade 3 or worse	Grade 3	Grade 4	Grade 5	Total grade 3 or worse	
Any toxicity	26 (9%)	260 (86%)	1 (<1%)	287 (95%)	20 (7%)	260 (87%)	1 (<1%)	281 (94%)	p=0·56
Non-haematological events†	197 (65%)	35 (12%)	1 <1%)	233 (77%)	187 (63%)	71 (24%)	1 (<1%)	259 (87%)	p=0·0024

* Comparison of grade 3 or higher toxicity.

† Any toxicity, excluding neutropenia, thrombocytopenia, anaemia, and leucopenia.
