# Supplementary material for: Comparison of MAPIE versus MAP in patients with a poor response to preoperative chemotherapy for newly diagnosed high-grade osteosarcoma (EURAMOS-1): an open-label, international, randomised controlled trial
Source: Lancet Oncol. 2016 Oct;17(10):1396–408. doi: 10.1016/S1470-2045(16)30214-5 (PMC5052459; doi:10.1016/S1470-2045(16)30214-5)
Supplement: Supplementary appendix [file mmc1.pdf]

THE LANCET Oncology

Supplementary appendix

This appendix formed part of the original submission and has been peer reviewed. We post it as supplied by the authors.

Supplement to: Marina NM, Smeland S, Bielack SS, et al. Comparison of MAPIE versus MAP in patients with a poor response to preoperative chemotherapy for newly diagnosed high-grade osteosarcoma (EURAMOS-1): an open-label, international, randomised controlled trial. *Lancet Oncol* 2016; published online Aug 25. http://dx.doi.org/10.1016/S1470-2045(16)30214-5.

SUPPLEMENTARY APPENDIX

Randomised Phase III Comparison of MAPIE vs MAP in patients with a Poor Response to pre-operative chemotherapy for newly-diagnosed high-grade osteosarcoma: results from the EURAMOS-1 trial

TABLE OF CONTENTS

=== SUPPLEMENTAL TABLES === 2

Table S1: Poor Responders randomisations, by country 3

Table S2: Reasons for non-randomisation, for patients with poor histological response 4

Table S3: Details of second malignancies 5

Table S4: Comparison of second malignancy between MAPIE and MAP arms 6

Table S5: Subgroups analyses, Restricted Mean Survival Time by arm, with difference, 95%CI, number of patients and events 7

Table S6: Less common postoperative treatment toxicities by allocated treatment, grade 3 and 4. 8

=== SUPPLEMENTAL FIGURES === 9

Figure S1: Treatment schedule 10

=== SUPPLEMENTAL LISTS === 11

List S1: EURAMOS-1 Investigators And Participants 12

List S2: Independent Members Of Independent Trial Oversight Committees 28

=== SUPPLEMENTAL TABLES ===

Table S1: Poor Responders randomisations, by country

Country	Number of randomised poor responders
USA	281
Germany	123
UK	72
Netherlands	26
Canada	18
Norway	17
Belgium	16
Switzerland	14
Austria	13
Sweden	9
Australia	8
Hungary	8
Denmark	4
Czech Republic	3
Finland	3
New Zealand	2
Ireland	1
**Total**	**618**

Table S2: Reasons for non-randomisation, for patients with poor histological response

Reason patient was not randomised	N	Percent
No consent	204	46.2%
Progression (local or distant)	111	25.1%
Late histology	45	10.2%
Wrong preoperative CT	31	7.0%
Other*	17	3.8%
No removal of metastases/ unresectable disease	16	3.6%
Not recovered from prior therapy	7	1.6%
Change of diagnosis	6	1.4%
Incomplete resection of primary tumour	5	1.1%
**Total**	**442**	**100%**

*Other reasons for non-randomisation were:

- not able to randomise within 35 days after definitive surgery (5 patients)
- patient choice (2 patients)
- physician choice (1 patient)
- site was too late to randomise the patient (2 patients)
- patient was registered as non-metastatic but actually had a metastatic disease. This was a stratification factor and it was not possible to amend it after registration (1 patient)
- surgery delayed (1 patient)
- unable to tolerate methotrexate (2 patients)
- uncertain radiological response to induction chemotherapy (1 patient)
- disagreement between local and central histology resulted in 3rd opinion on pathology which is likely to be non-randomisation due to late histology but not explicitly stated (1 patient),
- no response to induction chemotherapy (1 patient)

Table S3: Details of second malignancies

Second malignancy	ICD-O code	Time to SMN from randomisation	Received postoperative dose on trial	Number of doses on trial	Cytogenetic abnormality
**MAP arm**					
Acute myeloid leukemia	99203	1·2 years	MTX: 87·4 g/m^2<br>DOX: 295 mg/m^2<br>DDP: 224 mg/m^2	8 doses<br>4 doses<br>2 doses	
Myelodysplastic syndrome	99873	3.9 years	MTX: 76·5 g/m^2<br>DOX: 293 mg/m^2<br>DDP: 240 mg/m^2	7 doses<br>4 doses<br>2 doses	Monosomy 7
Squamous cell carcinoma	80703	6·4 years	MTX: 66·7 g/m^2<br>DOX: 300 mg/m^2<br>DDP: 242 mg/m^2	8 doses<br>4 doses<br>2 doses	
**MAPIE arm**					
Acute myeloid leukemia	9891-3	0·9 years	MTX: 61·5 g/m^2<br>DOX: 229 mg/m^2<br>DDP: 247 mg/m^2<br>I: 49·2 g/m^2<br>E: 1·4 g/m^2	5 doses<br>3 doses<br>2 doses<br>4 doses<br>3 doses	inv 11, 11q23 abnormality, chromosome 7
Acute myeloid leukemia	99203	1·2 years	MTX: 12·5 g/m^2<br>DOX: 75 mg/m^2<br>DDP: 118 mg/m^2<br>I: 0 g/m^2<br>E: 0 g/m^2	1 dose<br>1 dose<br>1 dose<br>0 doses<br>0 doses	t(9;11) 11q23
Acute myeloid leukemia	99203	2·4 years	MTX: 102·4 g/m^2<br>DOX: 318 mg/m^2<br>DDP: 220 mg/m^2<br>I: 61·0 g/m^2<br>E: 1·5 g/m^2	8 doses<br>4 doses<br>2 doses<br>5 doses<br>3 doses	
Acute myeloid leukemia	99203	3 years	MTX: 68·9 g/m^2<br>DOX: 298 mg/m^2<br>DDP: 239 mg/m^2<br>I: 51·5 g/m^2<br>E: 1·3 g/m^2	6 doses<br>4 doses<br>2 doses<br>5 doses<br>3 doses	Monosomy 7
Acute myeloid leukemia	99203	5·2 years	MTX: 12 g/m^2<br>DOX: 104 mg/m^2<br>DDP: 120 mg/m^2<br>I: 11·5 g/m^2<br>E: 0·36 g/m^2	1 dose<br>2 doses<br>1 dose<br>1 dose<br>1 dose	
Acute myeloid leukemia	99203	5·6 years	MTX: 94·1 g/m^2<br>DOX: 302 mg/m^2<br>DDP: 240 mg/m^2<br>I: 59·8 g/m^2<br>E: 1·5 g/m^2	8 doses<br>4 doses<br>4 doses<br>21 doses<br>15 doses	deletion Y chromosome
Ewing sarcoma (conventional osteosarcoma-osteoblastic, at diagnosis)	92603	5·7 years	MTX: 100 g/m^2<br>DOX: 304 mg/m^2<br>DDP: 241 mg/m^2<br>I: 60·5 g/m^2<br>E: 1·5 g/m^2	8 doses<br>4 doses<br>2 doses<br>5 doses<br>3 doses	t(6; 11; 22)-mediated EWSR1 gene rearrangement
Myelodysplasia	99873	2·1 years	MTX: 85·6 g/m^2<br>DOX: 147 mg/m^2<br>DDP: 113 mg/m^2<br>I: 14·0 g/m^2<br>E: 0·29 g/m^2	8 doses<br>4 doses<br>2 doses<br>6 doses<br>3 doses	
Myelodysplastic syndrome	99893	2·8 years	MTX: 71·0 g/m^2<br>DOX: 288 mg/m^2<br>DDP: 232 mg/m^2<br>I: 50·8 g/m^2<br>E: 0·98 g/m^2	6 doses<br>4doses<br>2 doses<br>5 doses<br>3 doses	
Thyroid carcinoma	80003	7·8 years	MTX: 92·5 g/m^2<br>DOX: 295 mg/m^2<br>DDP: 231 mg/m^2<br>I: 59·6 g/m^2<br>E: 1·5 g/m^2	8 doses<br>4 doses<br>2 doses<br>5 doses<br>3 doses	

Total ifosfamide dose includes doses at 14g/m^2/cycle and 9g/m^2/cycle.

Table S4: Comparison of second malignancy between MAPIE and MAP arms

	**Main event**<br>**Second malignancy**	**Competing event**<br>**Any other type of event**<br>(local recurrence, new metastases, progression of existing metastases, death or a combination of events)
**Cox model***	**Cause-specific HR (95%CI)**	**Cause-specific HR (95%CI)**
**Treatment**		
MAPIE vs MAP	3·24 (0·87-12·06), p=0·079	0·92 (0·73-1·15) p=0·46
**Fine-Gray model+**	**Sub-distribution HR (95%CI)**	**Sub-distribution HR (95%CI)**
**Treatment**		
MAPIE vs MAP	3·21 (0·87-11·85) p=0·081	0·91 (0·73-1·15) p=0·43

*proportionality of hazards tested: for the main event p=0·99, for the competing event p=0·0006
+proportionality not violated, tested with Schoenfeld-like residuals graph

Table S5: Subgroups analyses, Restricted Mean Survival Time by arm, with difference, 95%CI, number of patients and events

Exploratory subgroups	Restricted Mean Survival Time (months)				MAP		MAPIE	
	MAP	MAPIE	Difference (95% CI)	p-value	patients	events	patients	events
**Gender**								
Male	40·7	42·3	1·6 (-4·3; 7·6)	0·59	174	94	191	97
Female	46·6	47·0	0·4 (-6·2; 7·1)	0·90	136	59	117	57
**Age***								
Child	45·0	50·6	5·6 (-2·9; 14·1)	0·20	91	43	77	30
Adolescent	41·2	42·2	1·0 (-5·8; 7·8)	0·77	126	65	156	81
Adult	44·6	40·8	-3·7 (-11·8; 4·4)	0·37	93	45	75	43
**Site & location**								
Proximal femur/humerus	31·3	37·0	5·7 (-7·3; 18·7)	0·39	43	28	34	22
Other limb site	44·9	45·5	0·6 (-3·7; 4·8)	0·80	253	120	257	121
Axial skeleton	n/a	n/a	n/a	n/a	14	5	17	11
**Metastases status**								
Non metastatic¥	46·66	46·71	0·05 (-4·7; 4·8)	0·98	265	118	276	129
Metastatic	23·1	26·6	3·5 (-7·6; 14·5)	0·54	45	35	32	25
**Lung metastases**								
No¥	45·9	46·3	0·4 (-4·2; 5·0)	0·86	272	124	280	132
Yes	23·8	29·0	5·2 (-6·3; 16·8)	0·37	38	29	28	22
**Extra pulmonary metastases**								
No	43·7	45·2	1·5 (-2·9; 5·9)	0·50	302	147	299	147
Yes	n/a	n/a	n/a	n/a	8	6	9	7

¥possible metastases were combined with no metastases
*age:
- **Child**: male 0–12 yrs; female 0–11 yrs;
- **Adolescent**: male 13–17 yrs; female 12–16 yrs;
- **Adult**: male 18 yrs or older; female 17 yrs or older

Collins M, Wilhelm M, Conyers R, et al: Benefits and adverse events in younger versus older patients receiving neoadjuvant chemotherapy for osteosarcoma: findings from a meta-analysis. J Clin Oncol 31:2303-2312, 2013

Positive RMST difference MAPIE-MAP presents a longer mean time to first event for MAPIE patients

Table S6: Less common postoperative treatment toxicities by allocated treatment, grade 3 and 4.

Toxicity	MAP (n=301)				MAPIE (n=298)			
	Grade 3		Grade 4		Grade 3		Grade 4	
	N	%	N	%	N	%	N	%
Anorexia	2	1%	0	0%	2	1%	0	0%
Dehydration	3	1%	0	0%	1	0%	0	0%
Haemorrhage pulmonary - nose	2	1%	0	0%	2	1%	0	0%
Wound complication, non-infectious	2	1%	0	0%	2	1%	0	0%
Fatigue	1	0%	0	0%	2	1%	0	0%
Fracture	2	1%	0	0%	1	0%	0	0%
Liver dysfunction	2	1%	0	0%	0	0%	1	0%
Syndromes – other	1	0%	0	0%	2	1%	0	0%
Aphasia or dysarthria	2	1%	0	0%	0	0%	0	0%
Colitis	1	0%	0	0%	1	0%	0	0%
Constipation	2	1%	0	0%	0	0%	0	0%
Device/prosthesis	1	0%	0	0%	1	0%	0	0%
DVT/Thrombosis/Embolism	1	0%	1	0%	0	0%	0	0%
Hypotension	1	0%	0	0%	0	0%	1	0%
Lipase	0	0%	0	0%	1	0%	1	0%
Renal/genitourinary - other (specify)	0	0%	0	0%	0	0%	2	1%
Syncope (fainting)	0	0%	0	0%	2	1%	0	0%
Weight loss	1	0%	0	0%	1	0%	0	0%
Blood/bone marrow - other (specify)	0	0%	1	0%	0	0%	0	0%
Bone marrow, other (infarction)	0	0%	1	0%	0	0%	0	0%
Decubitus	0	0%	1	0%	0	0%	0	0%
Dermatology/skin - other (specify)	1	0%	0	0%	0	0%	0	0%
Dyspnoea	0	0%	0	0%	1	0%	0	0%
Dystonic reaction	1	0%	0	0%	0	0%	0	0%
Hemiparesis	1	0%	0	0%	0	0%	0	0%
Infection of the prosthesis	0	0%	0	0%	1	0%	0	0%
Infection with normal ANC - blood	0	0%	0	0%	1	0%	0	0%
Joint-effusion	0	0%	0	0%	1	0%	0	0%
Musculoskeletal - other (post-operative	0	0%	0	0%	1	0%	0	0%
Neuropathy: cranial - CN II	0	0%	1	0%	0	0%	0	0%
Pancreatitis	1	0%	0	0%	0	0%	0	0%
Pneumonia (metapneumo virus)	0	0%	0	0%	1	0%	0	0%
Prolonged QTc	1	0%	0	0%	0	0%	0	0%
Supraventricular tachycardia	0	0%	0	0%	1	0%	0	0%
Ventricular arrhythmia - ventricular fibrillation	0	0%	1	0%	0	0%	0	0%

Note: This table supplements Table 3. This includes only adverse events that were reported spontaneously by sites.

=== SUPPLEMENTAL FIGURES ===

Figure S1: Treatment schedule

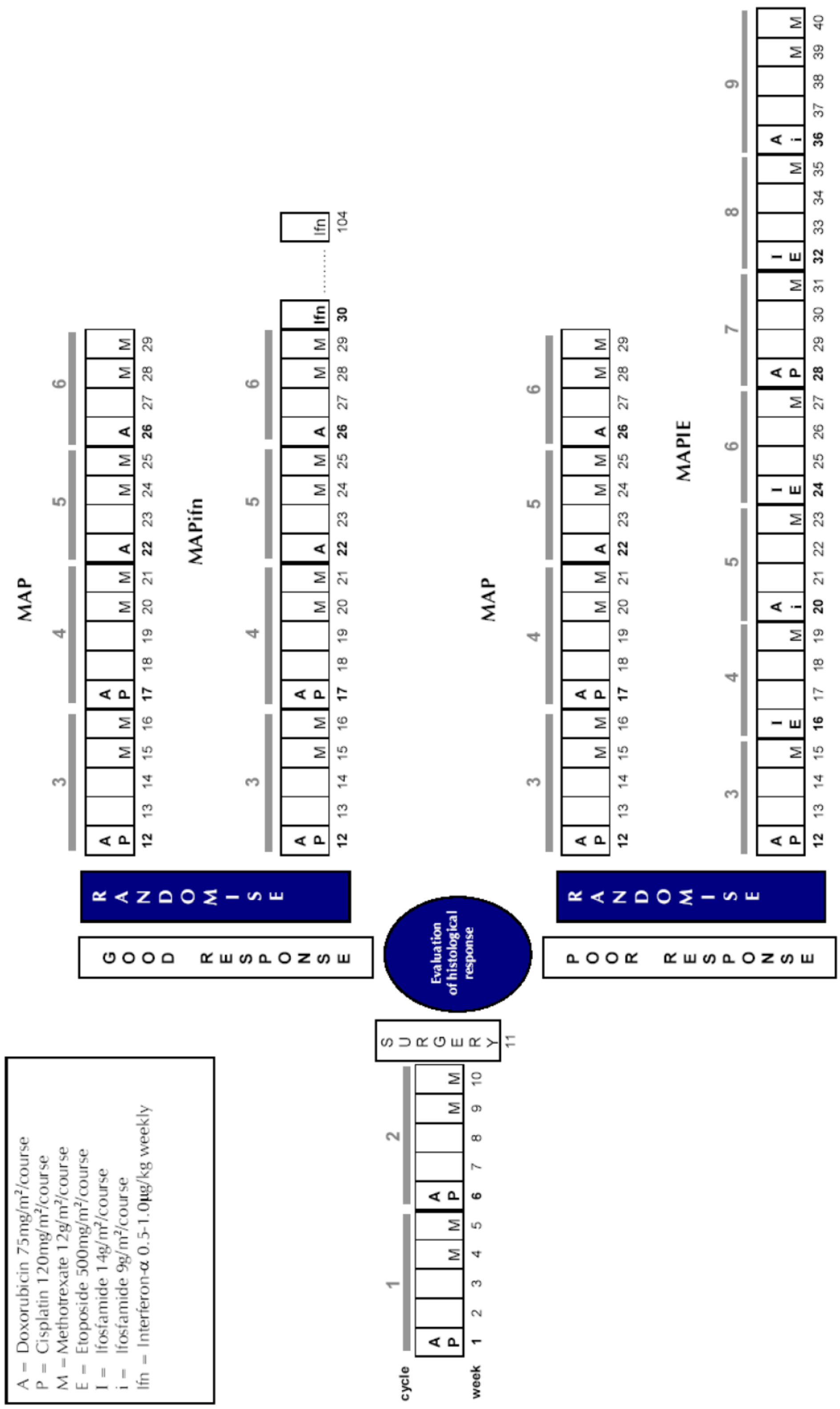

*Cisplatin and doxorubicin were administered at the same doses as given pre-operatively. Ifosfamide (high-dose) when combined with etoposide (3 post-operative cycles) was administered at 2.8g/m^2/day with mesna uroprotection (560 mg/m^2/dose) over 4 hours and etoposide at 100mg/m^2/day over 1 hour administered daily x 5 days. Two additional cycles of ifosfamide (standard dose) 3g/m^2/day with mesna uroprotection (600mg/m^2) daily for 3 days combined with standard doxorubicin. Growth factor support starting at least 24 hours was recommended after IE and DOX, I cycles, according to local practice e.g. filgrastim 5µg/kg or PEG-filgrastim 6mg subcutaneously.

=== SUPPLEMENTAL LISTS ===

List S1: EURAMOS-1 Investigators And Participants

EURAMOS-1: A randomised trial of the European and American Osteosarcoma Study Group to optimize treatment strategies for resectable osteosarcoma based on histological response to pre-operative chemotherapy is published in name of all the following collaborators:

:: S1.1. Children's Oncology Group (COG)

USA:
Advocate Hope Children's Hospital, Oak Lawn, IL (Dr. Ammar Hayani); Advocate Lutheran General Hospital, Park Ridge, IL (Dr. Jong Kwon); Albany Medical Center, Albany, NY (Dr. Vikramjit Kanwar); Alfred I duPont Hospital for Children, Wilmington, DE (Dr. Christopher Frantz); All Children's Hospital, St. Petersburg, FL (Dr. Gregory Hale); Ann and Robert H Lurie Children's Hospital of Chicago, Chicago, IL (Dr. David Walterhouse); Baptist Hospital of Miami, Miami, FL (Dr. Doured Daghistani); Baylor College of Medicine, Houston, TX (Dr. Lisa Bomgaars); BI-LO Charities Children's Cancer Center, Greenville, SC (Dr. Nichole Bryant); Bronson Methodist Hospital, Kalamazoo, MI (Dr. Jeffrey Lobel); Brooke Army Medical Center, Fort Sam Houston, TX (Dr. Della Howell); Brooklyn Hospital Center, New York, NY (Dr. Swayamprabha Sadanandan); Broward Health Medical Center, Fort Lauderdale, FL (Dr. Hector Rodriguez-Cortes); C S Mott Children's Hospital, Ann Arbor, MI (Dr. Rajen Mody); Cardinal Glennon Children's Medical Center, St Louis, MO (Dr. William Ferguson); Cardon Children's Medical Center, Mesa, AZ (Dr. Xiaxin Li); Carilion Clinic Children's Hospital, Roanoke, VA (Dr. Mandy Atkinson); Carolinas Medical Center/Levine Cancer Institute, Charlotte, NC (Dr. Joel Kaplan); Cedars-Sinai Medical Center, Los Angeles, CA (Dr. Fataneh (Fae) Majlessipour); Children's Healthcare of Atlanta - Egleston, Atlanta, GA (Dr. Howard Katzenstein); Children's Hospital and Medical Center of Omaha, Omaha, NE (Dr. Minnie Abromowitch); Children's Hospital and Research Center at Oakland, Oakland, CA (Dr. Carla Golden); Children's Hospital Central California, Madera, CA (Dr. Vonda Crouse); Children's Hospital Colorado, Aurora, CO (Dr. Kelly Maloney); Children's Hospital Medical Center of Akron, Akron, OH (Dr. Steven Kuerbitz); Children's Hospital of Alabama, Birmingham, AL (Dr. Alyssa Reddy); Children's Hospital of Los Angeles, Los Angeles, CA (Dr. Leo Mascarenhas); Children's Hospital of Philadelphia, Philadelphia, PA (Dr. Frank Balis); Children's Hospital of Pittsburgh of UPMC, Pittsburgh, PA (Dr. Arthur Ritchey); Children's Hospital-Main Campus, New Orleans, LA (Dr. Lolie Yu); Children's Hospitals and Clinics of Minnesota - Minneapolis, Minneapolis, MN (Dr. Bruce Bostrom); Children's Mercy Hospitals and Clinics, Kansas City, MO (Dr. Maxine Hetherington); Children's National Medical Center, Washington, DC (Dr. Jeffrey Dome); Childrens Hospital of Orange County, Orange, CA (Dr. Violet Shen); Childrens Hospital-King's Daughters, Norfolk, VA (Dr. Eric Lowe); Cincinnati Children's Hospital Medical Center, Cincinatti, OH (Dr. John Perentesis); City of Hope, Duarte, CA (Dr. Anna Pawlowska); Cleveland Clinic Foundation, Cleveland, OH (Dr. Margaret Thompson); Columbia University Medical Center, New York, NY (Dr. Alice Lee); Connecticut Children's Medical Center, Hartford, CT (Dr. Michael Isakoff); Cook Children's Medical Center, Fort Worth, TX (Dr. Mary Meaghan Granger); Midwest Children's Cancer Center, Milwaukee, WI (Dr. Michael Kelly); Miller Children's Hospital, Long Beach, CA (Dr. Amanda Termuhlen); Mission Hospitals Inc, Asheville, NC (Dr. Orren Beaty); Montefiore Medical Center - Moses Campus, Bronx, NY (Dr. Rosanna Ricafort); Morristown Medical Center, Morristown, NJ (Dr. Steven Halpern); Mount Sinai Medical Center, New York, NY (Dr. Birte Wistinghausen); Natalie W Bryant Cancer Center, Tulsa, OK (Dr. Gregory Kirkpatrick); Nationwide Children's Hospital, Colombus, OH (Dr. Mark Ranalli); Naval Medical Center - Portsmouth, Fort Sam Houston, TX (Dr. Della Howell); Naval Medical Center -San Diego, Fort Sam Houston, TX (Dr. Della Howell); Nemours Children's Clinic - Jacksonville, Jacksonville, FL (Dr. Eric Sandler); Nemours Children's Clinic - Pensacola, Pensacola, FL (Dr. Jeffrey Schwartz); Nemours Childrens Clinic - Orlando, Orlando, FL (Dr. Ramamoorthy Nagasubramanian); Nevada Cancer Research Foundation CCOP, Las Vegas, NV (Dr. Jonathan Bernstein); New York Medical College, Valhalla, NY (Dr. Mehmet Ozkaynak); Newark Beth Israel Medical Center, Newark, NJ (Dr. Peri Kamalakar); Ochsner Clinic Foundation, New Orleans, LA (Dr. Rajasekharan (Raj) Warrier); Oregon Health and Science University, Portland, OR (Dr. Linda Stork); Palmetto Health Richland, Columbia, SC (Dr. Ronnie Neuberg); Penn State Hershey Children's Hospital, Hershey, PA (Dr. Lisa McGregor); Phoenix Childrens Hospital, Phoenix, AZ (Dr. Jessica Boklan); Novant Health Presbyterian Medical Center, Charlotte, NC (Dr. Paulette Bryant); Primary Children's Medical Center, Salt Lake City, UT (Dr. Phillip Barnette); Providence Sacred Heart Medical Center and Children's Hospital, Spokane, WA (Dr. Judy Felgenhauer); Rady Children's Hospital - San Diego, San Diego, CA (Dr. William Roberts); Rainbow Babies and Childrens Hospital, Cleveland, OH (Dr. Yousif (Joe) Matloub); Randall Children's Hospital at Legacy Emanuel, Portland, OR (Dr. Janice Olson); Raymond Blank Children's Hospital, Des Moines, IA (Dr. Wendy Woods-Swafford); Rhode

Island Hospital, Providence, RI (Dr. Kevin Schwartz); Riley Hospital for Children, Indianapolis, IN (Dr. Robert Fallon); Rocky Mountain Hospital for Children-Presbyterian Saint Luke's Medical, Denver, CO (Dr. Jennifer Clark); Roswell Park Cancer Institute, Buffalo, NY (Dr. Martin Brecher); Saint Barnabas Medical Center (Newark Beth Israel Medical Center), Livingston, NJ (Dr. Stacey Rifkin-Zenenberg); Saint Christopher's Hospital for Children, Philadelphia, PA (Dr. Gregory Halligan); Saint John Hospital and Medical Center, Gross Pointe Woods, MI (Dr. Hadi Sawaf); Saint Joseph Children's Hospital of Tampa, Tampa, FL (Dr. Hardeo Panchoosingh); Saint Joseph's Regional Medical Center, Peterson, NJ (Dr. Mary Bonilla); Saint Jude Children's Research Hospital, Memphis, TN (Dr. Wayne Furman); Saint Jude Midwest Affiliate, Peoria, IL (Dr. Pedro De Alarcon); Saint Luke's Mountain States Tumor Institute, Boise, ID (Dr. Eugenia Chang); Saint Mary's Hospital, West Palm Beach, FL (Dr. Narayana Gowda); Saint Peter's University Hospital, New Brunswick, NJ (Dr. Stanley Calderwood); Saint Vincent Hospital, Green Bay, WI (Dr. John Hill); Saint Vincent Hospital and Health Services, Indianapolis, IN (Dr. Bassem Razzouk); San Jorge Children's Hospital, Santurce, PR (Dr. Luis Clavell); Sanford Medical Center-Fargo, Fargo, ND (Dr. Nathan Kobrinsky); Sanford USD Medical Center - Sioux Falls, Sioux Falls, ND (Dr. Kayelyn Wagner); Santa Barbara Cottage Hospital, Santa Barbara, CA (Dr. Daniel Greenfield); Scott and White Memorial Hospital, Temple, TX (Dr. Guy Grayson); Seattle Children's Hospital, Seattle, WA (Dr. Douglas Hawkins); Sinai Hospital of Baltimore, Baltimore, MD (Dr. Joseph Wiley); Southern California Permanente Medical Group, Los Angeles, CA (Dr. Robert Cooper); Southern Illinois University School of Medicine, Springfield, IL (Dr. Gregory Brandt); State University of New York Upstate Medical University, Syracuse, NY (Dr. Karol Kerr); Stony Brook University Medical Center, Stony Brook, NY (Dr. Robert Parker); T C Thompson Children's Hospital, Chattanooga, TN (Dr. Manoo Bhakta); Tampa General Hospital, Tampa, FL (Dr. Cameron Tebbi); Texas Tech University Health Science Center-Amarillo (Children' Hospital of San Antonio, San Antonio, TX (Dr. Osvaldo Regueira); The Children's Medical Center of Dayton (Dayton Children's Hospital), Dayton, OH (Dr. Emmett Broxson); The Steven and Alexandra Cohen Children's Medical Center of New York, New Hyde Park, NY (Dr. Arlene Redner); The Toledo Hospital/Toledo Children's Hospital, Toledo, OH (Dr. Dagmar Stein); The University of Arizona Medical Center-University Campus, Tuscon, AZ (Dr. Brenda Wittman); Tripler Army Medical Center, Fort Sam Houston, TX (Dr. Della Howell); UMDNJ - Robert Wood Johnson University Hospital (Rutgers Cancer Institute of New Jersey), New Brunswick, NJ (Dr. Richard Drachtman); University of Arkansas for Medical Sciences (Arkansas Children's Hospital), Little Rock, AK (Dr. David Becton); University of California Davis-Cancer Center, Sacramento, CA (Dr. Jay Michael Balagtas); University of California San Francisco Medical Center-Parnassus, San Francisco, CA (Dr. Robert Goldsby); University of Chicago, Chicago, IL (Dr. Susan Cohn); University of Florida, Gainesville, FL (Dr. William Slayton); University of Hawaii, Honolulu, HI (Dr. Robert Wilkinson); University of Illinois, Chicago, IL (Dr. Mary Schmidt); University of Iowa Hospitals and Clinics, Iowa City, IA (Dr. Ayman El-Sheikh); University of Kentucky, Lexington, KY (Dr. Martha Greenwood); University of Maryland Greenebaum Cancer Center, Baltimore, ND (Dr. Teresa York); University of Massachusetts Medical School, Worcester, MA (Dr. Christopher Keuker); University of Miami Miller School of Medicine-Sylvester Cancer Center, Miami, FL (Dr. Julio Barredo); University of Minnesota Medical Center-Fairview, Minneapolis, MN (Dr. Joseph Neglia); University of Mississippi Medical Center, Jackson, MS (Dr. Gail Megason); University of Missouri-Columbia (MUHC-Women and Children's Hospital), Columbia, MO (Dr. Thomas Loew); University of Nebraska Medical Center, Omaha, NE (Dr. Peter Coccia); University of New Mexico, Albuquerque, NM (Dr. Koh Boayue); University of North Carolina at Chapel Hill, Chapel Hill, NC (Dr. Stuart Gold); University of Oklahoma Health Sciences Center, Oklahoma City OK (Dr. Rene McNall-Knapp); University of Rochester, Rochester, NY (Dr. Jeffrey Andolina); University of South Alabama, Mobile, AL (Dr. Felicia Wilson); University of Texas Health Science Center, San Antonio, TX (Dr. Anne-Marie Langevin); University of Texas Southwestern Medical Center, Dallas, TX (Dr. Naomi Winick); University of Vermont, Burlington, VT (Dr. Alan Homans); University of Virginia, Charlottesville, VA (Dr. Kimberly Dunsmore); University of Wisconsin Hospital and Clinics, Madison, WI (Dr. Kenneth De Santes); Vanderbilt University, Nashville, TN (Dr. Haydar Frangoul); Wake Forest University Health Sciences, Winston-Salem, NC (Dr. Thomas McLean); Walter Reed National Military Medical Center, Fort Sam Houston, TX (Dr. Della Howell); Washington University School of Medicine, St Louis, MO (Dr. Robert Hayashi); Wayne State University, Detroit, MI (Dr. Zhihong Wang); Weill Medical College of Cornell University, New York, NY (Dr. Alexander Aledo); William Beaumont Hospital (Beaumont Children's Hospital-Royal Oak), Royal Oak, MI (Dr. Charles Main); Winthrop University Hospital, Mineola, NY (Dr. Mark Weinblatt); Yale University, New Haven, CT (Dr. Nina Kadan-Lottick); Covenant Children's Hospital, Lubbock, TX (Dr. Latha Prasannan); Dana-Farber Cancer Institute, Boston, MA (Dr. Carlos Rodriguez-Galindo); Dartmouth Hitchcock Medical Center, Lebanon, NH (Dr. Sara Chaffee); Dell Children's Medical Center of Central Texas, Austin, TX (Dr. Sharon Lockhart); Driscoll Children's Hospital, Corpus Christie, TX (Dr. M. Cris Johnson); Duke University Medical Center, Durham, NC (Dr. Susan Kreissman); East Carolina University, Greeneville, NC (Dr. Mauro Grossi); East Tennessee Childrens Hospital, Knoxville, TN (Dr. Ray Pais); Eastern Maine Medical Center, Brewer, ME (Dr. Sarah Fryberger); Georgia Regents University,

Augusta, GA (Dr. Colleen McDonough); Hackensack University Medical Center, Hackensack, NJ (Dr. Burton Appel); Helen DeVos Children's Hospital at Spectrum Health, Grand Rapids, MI (Dr. David Dickens); Hurley Medical Center, Flint, MI (Dr. Susumu Inoue); Inova Fairfax Hospital, Falls Church, VA (Dr. Marshall Schorin); Johns Hopkins University, Baltimore, MD (Dr. Kenneth Cohen); Kaiser Permanente-Oakland, Oakland, CA (Dr. Steven Bergstrom); Kosair Children's Hospital, Louisville, KY (Dr. Salvatore Bertolone); Lehigh Valley Hospital - Muhlenberg, Bethlehem, PA (Dr. Philip Monteleone); Loma Linda University Medical Center, Loma Linda, CA (Dr. Antranik Bedros); Loyola University Medical Center, Maywood, IL (Dr. Ricarchito Manera); Lucile Packard Children's Hospital Stanford University, Palo Alto, CA (Dr. Neyssa Marina); M D Anderson Cancer Center, Houston, TX (Dr. Najat Daw); Madigan Army Medical Center, Fort Sam Houston, TX (Dr. Della Howell); Maine Children's Cancer Program, Scarsborough, ME (Dr. Eric Larsen); Mark O Hatfield-Warren Grant Magnuson Clinical Center, Bethesda, MD (Dr. Crystal Mackall); Marshfield Clinic, Marshfield, WI (Dr. Michael McManus); Mary Bridge Children's Hospital and Health Center, Tacoma, WA (Dr. Robert Irwin); Massachusetts General Hospital Cancer Center, Boston, MA (Dr. Howard Weinstein); Mattel Children's Hospital UCLA, Los Angeles, CA (Dr. Pamela Kempert); Mayo Clinic, Rochester, MN (Professor Carola Arndt); Medical City Dallas Hospital, Dallas, TX (Dr. Carl Lenarsky); Medical University of South Carolina, Charleston, SC (Dr. Jacqueline Kraveka); MedStar Georgetown University Hospital, Washington, DC (Dr. Aziza Shad); Memorial Regional Hospital/Joe DiMaggio Children's Hospital, Hollywood, FL (Dr. Iftikhar Hanif); Memorial Sloan Kettering Cancer Center, New York, NY (Dr. Peter Steinherz); Memorial University Medical Center, Savannah, GA (Dr. J. Martin Johnston); Mercy Children's Hospital, Toledo, OH (Dr. Rama Jasty); Mercy Hospital Saint Louis, St Louis, MO (Dr. Robin Hanson); Methodist Children's Hospital of South Texas, San Antonio, TX (Dr. Jaime Estrada); Miami Children's Hospital, Miami, FL (Dr. Enrique Escalon); Michigan State University Clinical Center, East Lansing, MI (Dr. Renuka Gera); New York University Langone Medical Center, (Dr. Elizabeth Raetz)

Canada:

Alberta Children's Hospital, Calgary, AB (Dr. Douglas Strother); Allan Blair Cancer Centre, Regina, SK (Dr. Mansoor Haq); British Columbia Children's Hospital, Vancouver, BC (Dr. Caron Strahlendorf); Cancer Centre of Southeastern Ontario at Kingston General Hospital, Kingston, ON (Dr. Mariana Silva); CancerCare Manitoba, Winnipeg, MB (Dr. Rochelle Yanofsky); Centre Hospitalier Universitaire Sainte-Justine, Montreal, QC (Dr. Yvan Samson); Children's Hospital, London, ON (Dr. Anne Cairney); Children's Hospital of Eastern Ontario, Ottawa, ON (Dr. Jacqueline Halton); CHU de Quebec, Quebec, PQ (Dr. Bruno Michon); Saskatoon Cancer Centre, Saskatoon, SK (Dr. Christopher Mpofu); The Montreal Children's Hospital of the MUHC, Montreal, QC (Dr. Sharon Abish); University of Alberta Hospital, Edmonton, AB (Dr. Sunil Jayantilal Desai); Hospital for Sick Children, Toronto, ON (Dr. Ronald Grant); IWK Health Centre, Halifax, NS (Dr. Margaret Yhap); Janeway Child Health Centre, St John's, NF (Dr. Lisa Anne Goodyear); McMaster Children's Hospital at Hamilton Health Sciences, Hamilton, ON (Dr. Carol Portwine)

Australia:

Monash Medical Center-Clayton Campus, Clayton, VIC (Dr. Peter Downie); Princess Margaret Hospital for Children, Perth, WA. (Dr. Catherine Cole); Royal Children's Hospital, Parkville, VIC (Dr. Francoise Mechinaud); Royal Childrens Hospital-Brisbane, Brisbane, QLD (Dr. Helen Irving); Sydney Children's Hospital, Randwick, NSW (Dr. Draga Barbaric); The Children's Hospital at Westmead, Westmead, NSW (Dr. Geoffrey McCowage); Women's and Children's Hospital-Adelaide, North Adelaide, SA (Dr. Maria Kirby)

Switzerland:

Swiss Pediatric Oncology Group - Bern, Bern (Dr. Roland Ammann); Swiss Pediatric Oncology Group - Geneva, Geneva (Dr. Marc Ansari); Swiss Pediatric Oncology Group - Lausanne, Lausanne (Dr. Maja Beck Popovic)

New Zealand:

Christchurch Hospital, Christchurch (Dr. Michael Sullivan); Starship Children's Hospital, Auckland, (Dr. Teague)

Mexico:

Hospital Infantil de Mexico Federico Gomez, Mexico City (Dr. Aurora Medina-Sanson)

COG Panel Representatives and Additional Contributors
(*Group Representative)

Name	Role
Neyssa Marina	Chief Investigator<br>TMG member: Chief Investigator<br>National Coordinator<br>Oncology Panel
Mark Bernstein	Chief Investigator<br>TMG chair<br>National Coordinator<br>Oncology Panel
Caroline Wang,	TMG member: Research Coordinator
Justin Davis	TMG member: Research Coordinator
Possidonia Gontijo	TMG member: Research Coordinator
Mark Krailo	TMG Member: Statistician
	Statistics Panel
Allen Goorin	Oncology Panel
Paul Meyers	Oncology Panel
Lisa Teot*	Pathology review panel
Richard Gorlick*	Biological studies panel
Chand Khanna	Biological studies panel
Ching Lau,	Biological studies panel
Doug Letson*	Surgical Panel
Mark Gebhardt	Surgical Panel
Lor Randall	Surgical Panel
James Meyer	Radiology panel
Helen Nadel	Radiology panel
Paula Schomberg	Radiotherapy panel
Amy Barr	Pharmacy Panel

:: S1.2. Cooperative Osteosarkom Studiengruppe (COSS)

Austria:
Universitätsklinikum Graz, Klinik für Kinder- und Jugendheilkunde; Pädiatrische Hämatologie / Onkologie, Graz (Prof. Dr. C. Urban); Universitätsklinikum Innsbruck, Pädiatrie I; Department Kinder- und Jugendheilkunde, Innsbruck (Prof. Dr. B. Meister); Landes- Frauen- und Kinderklinik Linz, Kinder und Jugendheilkunde, Abteilung Onkologie, Linz (Prof. Dr. K. Schmitt); Landeskrankenhaus Salzburg, Universitätsklinikum der PMU, Abteilung Pädiatrische Hämatologie / Onkologie, Salzburg (Dr. N. Jones); St. Anna Kinderspital / CCRI, Vienna (Prof. Dr. L. Kager);

Czech Republic:
University Childrens Hospital Brno, Paediatric Oncology, Brno (Prof. Dr. P. Mudry); University Hospital MOTOL, Department of Paediatric Hematology und Oncology, Prague (Prof. Dr. J. Stary)

Germany:
Universitätsklinikum Aachen, Klinik für Kinder- und Jugendmedizin der Medizinischen Fakultät der RWTH, Bereich Pädiatrische Hämatologie / Onkologie, Aachen (Prof. Dr. U. Kontny); Klinikum Augsburg, I. Klinik für Kinder und Jugendliche, Augsburg (Prof. Dr. M. Frühwald); Sozialstiftung Bamberg, Klinikum am Bruderwald, Zentrum Innere Medizin, Bamberg (PD Dr. R Repp); Helios Klinikum Berlin-Buch, Klinik für Kinder und Jugendmedizin, Spezielle Hämatologie und Onkologie, Berlin (Prof. Dr. L. Schweigerer); Helios Klinikum Berlin-Buch, Sarkomzentrum Berlin-Brandenburg, Berlin (PD Dr. P. Reichardt); Charité - Universitätsmedizin Berlin, Campus Virchow Klinikum, Medizinische Klinik mit Schwerpunkt Hämatologie, Onkologie und Tumorimmunologie, Berlin (Dr. P. Thuss-Patience); Charité - Universitätsmedizin Berlin, Campus Virchow-Klinikum, Klinik für Pädiatrie mit Schwerpunkt Hämatologie und Onkologie, Berlin (Prof. Dr. A. Eggert); Evangelisches Krankenhaus Bielefeld, Kinderklinik, Abteilung für Pädiatrische Hämatologie und Onkologie, Bielefeld (Dr. N. Jorch); Universitätsklinikum Bonn, Zentrum für Kinderheilkunde; Pädiatrische Hämatologie und Onkologie, Bonn (Prof. Dr. D. Dilloo); Klinikum Braunschweig, Pädiatrische Hämatologie / Onkologie, Braunschweig (Dr. W. Eberl); Klinikum Bremen – Mitte / Prof.-Hess-Kinderklinik, Bremen (Prof. Dr. A. Pekrun); Evangelisches Diakonie-Krankenhaus, Abteilung Hämatologie / Internistische Onkologie, Bremen (Prof. Dr. R. Trappe); Klinikum Chemnitz, Klinik für Kinder- und Jugendmedizin; Abteilung Hämatologie / Onkologie, Chemnitz (Dr. A. Hofmann); Klinikum Dortmund, Medizinische Klinik Mitte, Dortmund (Dr. S. Bäumer); Klinikum Dortmund , Klinik für Kinder- und Jugendmedizin; Abteilung Pädiatrische Onkologie / Hämatologie, Dortmund (Prof. Dr. D. Schneider); Universitätsklinikum Dresden, Klinik und Poliklinik für Kinder- und Jugendmedizin, Dresden (Prof. Dr. M. Suttorp); Universitätsklinikum Dresden, Medizinische Klinik I; Fachbereich Onkologie, Dresden (PD Dr. G. Folprecht); Universitätsklinikum Düsseldorf, Klinik für Kinder-Onkologie, -Hämatologie und Klinische Immunologie, Düsseldorf (Prof. Dr. A. Borkhardt); Helios Klinikum Erfurt, Klinik für Kinder- und Jugendmedizin; Pädiatrische Onkologie / Hämatologie, Erfurt (Prof. Dr. A. Sauerbrey); Universitätsklinikum Erlangen, Kinder- und Jugendklinik; Abteilung Hämatologie / Onkologie, Erlangen (Prof. Dr. M. Metzler); Universitätsklinikum Erlangen, Medizinische Klinik 5 - Hämatologie und Intern. Onkologie, Erlangen (Prof. Dr. B. Spriewald); Universitätsklinikum Essen, Klinik für Kinderheilkunde III; Abteilung für Pädiatrische Hämatologie, Onkologie und Endokrinologie, Essen (Prof. Dr. D. Reinhardt); Universitätsklinikum Frankfurt, Klinik für Kinder- und Jugendmedizin, Schwerpunkt Onkologie, Hämatologie und Hämostaseologie, Frankfurt (Prof. Dr. Th. Klingebiel); Universitätsklinikum Freiburg, Zentrum für Kinder- und Jugendmedizin; Klinik IV: Pädiatrische Hämatologie und Onkologie, Freiburg (Prof. Dr. Ch. Niemeyer); Universitätsklinikum Gießen und Marburg, Standort Gießen, Zentrum für Innere Medizin IV, Gießen (Prof. Dr. M. Rummel); Universitätsklinikum Gießen und Marburg, Standort Gießen, Kinderheilkunde und Jugendmedizin, Pädiatrische Hämatologie und Onkologie, Gießen (Prof. Dr. W. Wößmann); Universitätsklinikum Göttingen, Abteilung Pädiatrie I mit Schwerpunkt Hämatologie und Onkologie, Göttingen (Prof. Dr. Ch. Kramm); Universitätsklinikum Greifswald, Klinik und Poliklinik für Kinder- und Jugendmedizin; Abteilung Pädiatrische Onkologie und Hämatologie, Greifswald (Prof. Dr. H. Lode); Universitätsklinikum Halle, Innere Medizin IV, Abteilung Hämatologie / Onkologie, Halle (Dr. H.-H. Wolf); Universitätsklinikum Halle, Klinik für Kinder und Jugendmedizin; Abteilung Hämatologie / Onkologie, Halle / Saale (Prof. Dr. Ch. Mauz-Körholz); Universitätsklinikum Hamburg-Eppendorf, Abteilung für Pädiatrische Hämatologie und Onkologie, Hamburg (Dr. W. Hassenpflug); Universitätsklinikum Hamburg-Eppendorf, Medizinische Klinik II; Abteilung Onkologie / Hämatologie, Hamburg (Prof. Dr. C. Bokemeyer); Medizinische Hochschule Hannover, Kinderheilkunde; Pädiatrische Onkologie und Hämatologie, Hannover (Prof. Dr. A. Beilken); Medizinische Hochschule Hannover, Zentrum Innere Medizin; Abteilung Hämatologie / Onkologie, Hannover (Prof. Dr. Ch. Reuter); Universitätsklinikum Heidelberg, Innere Medizin V: Hämatologie, Onkologie und Rheumatologie, Heidelberg

(Prof. Dr. G. Egerer); Universitätsklinikum Heidelberg, Zentrum für Kinder- und Jugendmedizin; Abteilung III, Hämatologie / Onkologie / Immunologie, Heidelberg (Dr. W. Behnisch); SLK Kliniken Heilbronn, Klinik für Kinder und Jugendmedizin (Kinderonkologie), Heilbronn (Dr. M. Streiter); Gemeinschaftskrankenhaus Herdecke, Klinik für Kinder und Jugendmedizin, Herdecke (PD Dr. A. Längler); Universitätsklinikum des Saarlandes, Klinik für Kinder- und Jugendheilkunde; Pädiatrische Hämatologie / Onkologie, Homburg (Prof. Dr. N. Graf); Universitätsklinikum des Saarlandes, Klinik für Innere Medizin I, Homburg (Prof. Dr. M. Pfreundschuh); Universitätsklinikum Jena, Klinik für Kinder- und Jugendmedizin, Allg. Pädiatrie, Hämatologie, Onkologie und Immunologie, Jena (Dr. B. Gruhn); Universitätsklinikum Schleswig-Holstein, Campus Kiel, II. Medizinische Klinik und Poliklinik, Kiel (Prof. Dr. M. Kneba); Universitätsklinikum Schleswig-Holstein, Campus Kiel, Klinik für Allgemeine Pädiatrie, Pädiatrische Onkologie / Hämatologie, Kiel (Prof. Dr. M. Schrappe); Gemeinschaftsklinikum Mittelrhein Kemperhof, Klinik für Pädiatrische Hämatologie und Onkologie, Koblenz (Dr. R. Ferrari); Universitätsklinikum Köln, Zentrum für Kinderonkologie und Hämatologie, Köln (Prof. Dr. T. Simon); Universitätsklinikum Köln, Klinik I für Innere Medizin, Köln (Prof. Dr. J-M. Chemnitz); Helios Klinikum Krefeld, Kinderklinik, Krefeld (Dr. T. Imschweiler); Universitätsklinikum Leipzig, Abteilung für Pädiatrische Onkologie, Hämatologie, und Hämostaseologie, Leipzig (Prof. Dr. H. Christiansen); Universitätsklinikum Leipzig, Medizinische Klinik II (Hämatologie / Onkologie), Leipzig (Prof. Dr. D. Niederwieser); Universitätsklinikum Schleswig-Holstein, Campus Lübeck, Klinik für Kinder und Jugendmedizin, Pädiatrische Onkologie und Hämatologie, Lübeck (PD Dr. M. Lauten); St. Marien u. St. Annastiftskrankenhaus Ludwigshafen, Ludwigshafen (B. Selle); Universitätsklinikum Magdeburg, Arbeitsbereich Pädiatrische Hämatologie und Onkologie, Magdeburg (Prof. Dr. P. Vorwerk); Universitätsmedizin Mainz, Zentrum für Kinder- und Jugendmedizin; Pädiatrische Hämatologie / Onkologie, Mainz (PD Dr. J. Faber); Universitätsklinikum Gießen und Marburg; Standort Marburg, Kinderklinik; Abteilung Hämatologie / Onkologie, Marburg (Dr. B. Schütz); Johannes Wesling Klinikum Minden, Klinik für Kinder- und Jugendmedizin, Pädiatrische Onkologie und Hämatologie, Minden (Prof. Dr. B. Erdlenbruch); Klinikum München Schwabing, Kinderklinik der TU München, Klinik und Poliklinik für Kinder- und Jugendmedizin, München (PD Dr. I. Teichert von Lüttichau); Klinikum der LMU; Dr. von Haunersches Kinderspital, Zentrum für Pädiatrische Hämatologie und Onkologie, München (PD Dr. I. Schmid); Universitätsklinikum Großhadern, Medizinische Klinik III, München (PD Dr. L. Lindner); Klinikum rechts der Isar der TU München, III. Medizinische Klinik und Poliklinik; Abteilung Hämatologie / Onkologie, München (Dr. S. Lorenzen); Universitätsklinikum Münster, Medizinische Klinik und Poliklinik; Innere Medizin A, Münster (Prof. Dr. W. Berdel); Universitätsklinikum Münster, Klinik und PoliKlinik für Kinder und Jugendmedizin - Pädiatrische Hämatologie und Onkologie, Münster (Prof. Dr. H. Jürgens); Diakonie Neuendettelsau / Cnopf´sche Kinderklinik, Abteilung Hämatologie / Onkologie, Nürnberg (Prof. Dr. W. Scheurlen); Klinikum Nürnberg Nord, Klinik für Innere Medizin 5; Schwerpunkt Onkologie / Hämatologie, Nürnberg (Prof. Dr. M. Wilhelm); Klinikum Oldenburg , Zentrum für Kinder- und Jugendmedizin; Elisabeth-Kinderkrankenhaus, Oldenburg (Prof. Dr. H. Müller); Brüderkrankenhaus St. Josef Paderborn, Klinik für Hämatologie und Onkologie, Paderborn (Dr. T. Gaska); Universitätsklinikum Regensburg, Klinik für Kinder- und Jugendmedizin; Abteilung Pädiatrische Hämatologie / Onkologie, Regensburg (Prof. Dr. S. Corbacioglu); Krankenhaus Barmherzige Brüder Regensburg, Klinik für Onkologie und Hämatologie, Regensburg (Prof. Dr. J. Braess); Universitätsklinikum Regensburg, Klinik und Poliklinik für Innere Medizin III, Regensburg (Dr. Ch. Hart); Universitätsklinikum Rostock, Klinik für Kinder- und Jugendmedizin; Abteilung Hämatologie / Onkologie, Rostock (Prof. Dr. C. F. Classen); Diakoniekrankenhaus Rotenburg, I. Medizinische Klinik, Rotenburg (Dr. F. Heits); Helios Klinikum Schwerin, Klinik für Kinder- und Jugendmedizin, Schwerin (Dr. Kasbohm); Asklepios Klinik St. Augustin, Abteilung für Kinder- und Jugendheilkunde Hämatologie / Onkologie, St. Augustin (Dr. H. Reinhard); Klinikum Stuttgart - Olgahospital, Zentrum für Kinder-, Jugend- und Frauenmedizin, Pädiatrie 5 (Onkologie, Hämatologie, Immunologie), Stuttgart (Prof. Dr. S. Bielack); Marienhospital Stuttgart, Zentrum für Innere Medizin III, Stuttgart (Prof. Dr. Denzlinger); Klinikum Stuttgart - Krankenhaus Bad Cannstatt, Klinik für Allgemeine Innere Medizin, Gastroenterologie, Hepatologie und Internistische Onkologie, Stuttgart (Prof. Dr. T. Andus); Klinikum Mutterhaus der Borromäerinnen, Kinder- und Jugendmedizin, Trier (Dr. S. Weis); Klinikum Mutterhaus der Borromäerinnen, Innere Medizin 1 (Onkologisches Zentrum), Trier (Dr. R. Mahlberg); Krankenhaus der Barmherzigen Brüder, I. Med. Abteilung Hämato-Onkologie, Trier (Prof. Dr. H. Kirchen); Universitätsklinikum Tübingen, Innere Medizin II; Zentrum für Weichteilsarkome/GIST und Knochentumore, Tübingen (Prof. Dr. H.-G. Kopp); Universitätsklinikum Tübingen, Abteilung Kinderheilkunde I; Hämatologie / Onkologie, Tübingen (Prof. Dr. R. Handgretinger); Universitätsklinikum Ulm, Klinik für Kinder- und Jugendheilkunde; Pädiatrische Hämatologie / Onkologie, Ulm (Prof. Dr. M. Debatin); Universitätsklinikum Ulm, Zentrum für Innere Medizin, Klinik für Innere Medizin III, Ulm (Dr. R. Mayer-Steinacker); Bundeswehrkrankenhaus Ulm, Abteilung I Innere Medizin, Ulm (Dr. T. Mayer); Universitätsklinikum Würzburg, Pädiatrische Hämatologie und Onkologie, Würzburg (Prof. Dr. P.-G. Schlegel);

Hungary:
Állami Egészségügyi Központ, Onkologiai Osztály, Budapest (Dr. Z Papai); 2nd Department of Paediatrics, Semmelweis University, Budapest (Dr. G. Kovacs)

Switzerland:
Kantonspital Aarau, Klinik für Kinder- und Jugendliche; Abteilung Pädiatrische Onkologie, Aarau (Dr. R. Angst); Universitätsspital Basel, Klinik für Kinder- und Jugendliche; Abteilung Hämatologie / Onkologie, Basel (Prof. Dr. T. Kühne); Universitäts-Kinderspital Zürich, Poliklinik Onkologie, Zürich (Prof. Dr. F. Niggli); Orthop. Universitätsklinik Balgrist, Zürich (Prof. Dr. B. Fuchs); Stadtspital Triemli, Institut für Med. Onkologie und Hämatologie, Zürich (Prof. Dr. Schmid);

:: COSS Panel Representatives and Additional Contributors
(*Group Representative)

Name	Role
Stefan Bielack	Chief Investigator<br>TMG member: Chief Investigator<br>National Coordinator: Germany<br>Oncology panel: Paediatric oncology
Beate Kempf-Bielack	TMG Member: Study Physician
Miriam Wilhelm	TMG Member: Study Physician
Dorothe Carrle	TMG Member; Study Physician
Katja Zils	TMG Member: Study Physician
Joachim Gerss	TMG Member: Statistician
Matthias Kevric	TMG Member: Data Manager
Benjamin Sorg	TMG Member: Data Manager
Claudia Wurster	Secretary
Feven Assefaw	Secretary
Lynn Hazlewood	Secretary
Leo Kager	National Coordinator: Austria<br>Oncology panel: Paediatric oncology
Zsuzsanna Papai	National Coordinator: Hungary
Thomas Kühne	National Coordinator: Switzerland
Hubert Mottl	National Coordinator: Czech Republic
Thomas Kühne	Oncology panel: Paediatric oncology
Peter Reichardt	Oncology panel: Medical oncology
Zsuzsanna Papai	Oncology panel: Medical oncology
Udo Kontnyl	Oncology panel: Paediatric oncology
W. Hassenpflug	Oncology panel: Paediatric oncology
Norbert Graf	Oncology panel: Paediatric oncology
Michaela Nathrath	Oncology panel: Paediatric oncology<br>Biological Studies Panel
Wolfgang E. Berdel	Oncology panel: Medical oncology
Heribert Jürgens	Oncology panel: Paediatric oncology

Name	Role
Regine Meyer-Steinacker	Oncology panel: Medical oncology
Gernot Jundt*	Pathology review panel
Andreas Schulz	Pathology review panel
Günter Delling	Pathology review panel
Mathias Werner	Pathology review panel
Albert Roessner	Pathology review panel
Gabriele Köhler<br>*(associate:* Ch. Dierkes)	Pathology review panel
Horst Bürger	Pathology review panel
Susanna Lang<br>(*associate:* Irene Sulzbacher)	Pathology review panel
Arthur R. von Hochstetter	Pathology review panel
Gabriela Arato	Pathology review panel
Gernot Jundt*	Biological Studies Panel
W. Hassenpflug	Biological Studies Panel
Rainhard Windhager*<br>(*associate:* Martin Dominkus)	Surgical Panel
Per-Ulf Tunn	Surgical Panel
Miklos Szendroi<br>(*associate:*. Imre Antal)	Surgical Panel
Detlev Branscheid	Surgical Panel: Thoracic Surgery representative
Godehard Friedel	Surgical Panel: Thoracic surgery representative
Ulrich Heise	Surgical Panel
Volker Ewerbeck	Surgical Panel
H. Roland Dürr	Surgical Panel
Axel Hillmann	Surgical Panel
Georg Gosheger<br>(ass*ociate:* Jendrik Hardes)	Surgical Panel
G. Ulrich Exner	Surgical Panel
Joachim Sciuk	Radiology Panel: Nuclear Medicine
Knut Helmke	Radiology Panel: Diagnostic Radiology
Peter Winkler<br>(*associate: Thekla v. Kalle)*	Radiology Panel: Diagnostic Radiology
Reiner Maas	Radiology Panel: Diagnostic Radiology
Christiane Franzius	Radiology Panel: Nuclear Medicine
Joachim Gerss	Statistics Panel
Rudolf Schwarz	Radiotherapy Panel
Beate Timmermann	Radiotherapy Panel: Particle Therapy
Elvira Ahlke & Hedwig Kolve	Pharmacy Panel

:: S1.3. European Osteosarcoma Intergroup (EOI)

UK:

University College Hospital, London (Prof. Jeremy Whelan); Royal Hospital for Sick Children, Edinburgh (Dr. Hamish Wallace); Birmingham Childrens Hospital, Birmingham (Dr. Bruce Morland); Sheffield Children's Hospital, Sheffield (Dr. Vicki Lee, Dr. Mary Gerrard); Christie Hospital, Manchester (Dr. Bernadette Brennan); Royal Manchester Children's Hospital, Manchester (Dr. Bernadette Brennan); Addenbrooke's Hospital, Cambridge (Dr. Amos Burke); Royal Marsden Hospital, Sutton (Dr. Julia Chisholm, Prof. Kathy Pritchard-Jones); Royal Hospital for Sick Children, Glasgow (Dr. Milind Ronghe); Beatson West of Scotland Cancer Centre, Glasgow (Dr. Jeff White); Royal Aberdeen Childrens Hospital, Aberdeen (Dr. Derek King); Queens Medical Centre, Nottingham (Prof. David Walker); St James University Hospital , Leeds (Dr. Susan Picton, Dr. Ian Lewis); Children's Hospital for Wales, Cardiff (Dr. Heidi Traunecker); Royal Orthopaedic Hospital, Birmingham (Dr. David Peake); Royal Victoria Infirmary, Newcastle (Dr. Juliet Hale); Nottingham University Hospitals, City Campus, Nottingham (Dr. Ivo Hennig, Dr. Michael Sokal); Southampton General Hospital, Southampton (Dr. Gary Nicolin); Bristol Royal Hospital for Children, Bristol (Prof. Mike Stevens); Ninewells Hospital, Dundee (Dr. Michelle Ferguson, Prof. Elaine Rankin); Weston Park Hospital, Sheffield (Prof. Penella Woll); Leicester Royal Infirmary, Leicester (Dr. Johannes Visser); Clatterbridge Centre for Oncology, Liverpool (Dr. Peter Clark, Dr. Nasim Ali); Churchill Hospital, Oxford (Prof. Bass Hassan); Royal Liverpool Childrens Hospital, Liverpool (Dr. Barry Pizer, Dr. Heather McDowell); Bristol Haematology & Oncology Centre, Bristol (Prof. Mike Stevens); Newcastle General Hospital, Newcastle (Dr. Mark Verrill)

Netherlands:

Leiden University Medical Center, Leiden (Prof. Hans Gelderblom, Dr. Jakob Anninga); Emma's Children's Hospital / AMC Amsterdam, Amsterdam (Dr. Henk Van Den Berg); Radboud University Nijmegen Medical Centre, Nijmegan (Prof. Hoogerbrugge); University Medical Center Groningen, Groningen (Prof. WA Kamps, Dr. ESJM De Bont)

Belgium:

University Hospital Ghent, Ghent (Dr. Catharina Dhooge); UZ Brussels, Brussels (Dr. An Van Damme, Dr. Jutte Van der Werff ten Bosch); Jules Bordet Institute, Brussels (Dr. Thierry Gil); Universitaire Ziekenhuizen Leuven, Leuven (Dr. Marleen Renard); Cliniques Universitaires Saint-Luc, Brussels (Prof. Benedicte Brichard); Hopital Universitaire des Enfants, Brussels (Dr. Sophie Huybrechts)

Ireland:

Our Lady's Children's Hospital, Dublin (Dr. Michael Capra)

:: EOI Panel Representatives and Additional Contributors
(*Group Representative)

Name	Role
Jeremy Whelan	Chief Investigator<br>National Coordinator: United Kingdom<br>Oncology Panel<br>TMG Member: Chief Investigator
Sandra J Strauss	Study Physician
Nicola Joffe	TMG Member<br>Clinical Project Manager
Sue Fleck	TMG Member<br>Clinical Project Manager
Karen Sanders	TMG Member<br>Clinical Project Manager<br>Data Manager
Barbara Uscinska	TMG Member<br>Clinical Project Manager
James Pickering	TMG Member<br>Trial Manager<br>Data Manager
Gintare Rutkauskaite	TMG Member<br>Trial Manager
Aurelie Faysse	TMG Member<br>Trial Manager
Sarah Beall	TMG Member<br>Trial Manager
Beth May	TMG Member<br>Trial Manager
Anne Holliday	TMG Member<br>Trial Manager
Monique Tomiczek	TMG Member<br>Trial Manager<br>Data Manager
Matthew Sydes	TMG Member<br>Trial Statistician<br>Statistics panel
Gordana Jovic	TMG Member<br>Statistician
Rachel Jinks	TMG Member<br>Statistician
Jane Hook	TMG Member<br>Study Physician
Leonardo Trani	TMG Member<br>Study Physician
Martha Perisoglou	TMG Member<br>Study Physician

Name	Role
Catharina Dhooge (Claeys)	National Coordinator: Belgium<br>Oncology Panel
Michael Capra	National Coordinator: Ireland
Hans Gelderblom	National Coordinator: Netherlands<br>Oncology Panel
Jakob Anninga	National Coordinator: Netherlands<br>Oncology Panel
Bruce Morland	Oncology Panel
Ian Lewis	Oncology Panel
Pancras Hogendoorn*	Pathology review panel<br>Biological Studies Panel
Judith V.M.G. Bovee	Pathology review panel
J. Bras	Pathology review panel
Adrienne Flanagan	Pathology review panel
Elaine MacDuff	Pathology review panel
Robin Reid	Pathology review panel
Petra Dildey	Pathology review panel
David E Hughes	Pathology review panel
Esther Hauben	Pathology review panel
Chas Mangham	Pathology review panel
Anne Marie Cleton Jansen	Biological Studies panel
Rob Grimer*	Surgical panel
Steve Cannon	Surgical panel
Anthony Taminiau	Surgical panel
Michael Gebhart	Surgical panel
Mark Davies	Radiology panel
Paul O'Donnell	Radiology panel
William Ramsden	Radiology panel
H.J. van der Woude	Radiology panel
Koenraad Verstraete	Radiology panel
Anna Cassoni	Radiotherapy panel
Denise Blake	Pharmacy panel

:: S1.4. Scandinavian Sarcoma Group (SSG)

Norway:

Oslo University Hospital (Dr. Kirsten Sundby Hall, Dr Heidi Glosli); Bergen University Hospital (Dr. Odd Monge); Trondheim University Hospital (Dr. Erling Moe)

Sweden:

Umeå University Hospital (Dr. Ulf Hjalmars (Children), Dr. Beatrice Malmer, Dr. Kjell Johansson (Children)); Linköping University Hospital (Dr. Najme Wall, Dr. Maria Östlund, Dr. Mikael Behrendtz); Gothenburg Sahlgrenska University Hospital (Dr. Lina Hansson, Dr. Gustaf Österlundh (Children), Dr. Monika Sender, Dr. Katarina Engström); Lund University Hospital, Oncologic dept. (Dr. Mikael Eriksson, Dr. Lars Hjorth (Children)); Akademiska sjukhuset, Uppsala University Hospital (Dr. Ingela Turesson, Dr. Gustaf Ljungman (Children)); Karolinska University Hospital, Stockholm (Dr. Elisabet Lidbrink, Dr. Cecilia Petersen (Children), Dr. Mikael Szeps, Dr. Annika Folin, Dr. Christina Linder-Stragliotto, Dr. Jonas Karlén (Children), Dr. Åke Jacobson (Children))

Finland:

Tampere University Hospital (Dr. Tuula Lehtinen); Helsinki University Central Hospital (Dr. Maija Tarkkanen); Turku University Hospital (Dr. Paula Lindholm)

Denmark:

Aarhus University Hospital (Dr. Akmal Safwat (Adult), Dr. Ole Steen Nielsen, Dr. Henrik Hasle (Children)); Copenhagen Rigshospital (Dr.Catherine Rechnitzer)

SSG Panel Representatives and Additional Contributors

(*Group Representative)

**Name**	**Role**
Sigbjørn Smeland	Chief Investigator<br>TMG Member: Chief Investigator<br>National Coordinator: Norway<br>Oncology Panel
Maria Rejmyr-Davis	TMG Member: Data Manager
Eva-Mari Olofsson	TMG Member: Research Administrator
Elisabeth Johansson	TMG Member: Data Manager
Linda Werner-Hartman	TMG Member: Statistician
Oskar Hagberg	Statistician
Karolina Carlsson	Statistician
Viktoria Samuelsson	Statistician
Anna Bladtröm	Statistician
Thor Alvegård	TMG Member: Data Manager Supervisor
Jeanette Ceberg	Monitor
Christina Danewid	Monitor
Mercedes Marotta	Data Systems Engineer
Mona Malström	CRF Designer

Name	Role
Ole Sten Nielsen	National Co-ordinator: Denmark
Maija Tarkkanen	National Co-ordinator: Finland
Oskar Johansson	National Co-ordinator: Iceland
Mikael Eriksson	National Co-ordinator: Sweden
	Oncology panel
Thomas Wiebe	Oncology Panel
Åke Jakobson	Oncology Panel
Maija Tarkkanen	Oncology Panel
Ole Steen Nielsen	Oncology Panel
Tom Böhling*	Pathology review panel
Lars-Gunnar Kindblom*	Pathology review panel
Henryk Domanski	Pathology review panel
Bodil Bjerkehagen	Pathology review panel
Johan Wejde	Pathology review panel
Ola Myklebost	Biological Studies panel
Nils Mandahl	Biological Studies panel
Sakari Knuutila	Biological Studies panel
Otte Brosjö	Surgical panel
Ingeborg Taksdal*	Radiology panel
Veli Söderlund	Radiology panel
Øyvind Bruland	Radiotherapy panel
Linda Werner-Hartman	Statistics panel
Tor Skärby	Pharmacy panel

S1.5. Members of EURAMOS Intergroup Safety Desk (EISD)

Trude Butterfaß-Bahloul	TMG Member: Clinical Research Associate SAE, Safety Desk Manager
Heidi Oellers	TMG Member: Monitoring/Auditing
Marc Urban	TMG Member: Monitoring/Auditing
Karl-Friedrich Lukat	Clinical Research Associate SAE, Safety Desk Manager
Melanie Langeleist	Safety Desk Assistant
Dorothee Hülser	Safety Desk Assistant
Gudrun Würthwein	Data Management of Safety Database
Sonja Baier	Data Management of Safety Database
Attyla Drabik	Safety Desk Manager
Charlotte Young	Safety Desk Manager
Kirsten Werner	Safety Desk Manager
Andrea Paneitz	Safety Desk Manager
Ruth Wagner	Safety Desk Manager
Eva Grünewald	Safety Desk Manager
Christiana Rohde-Osei	Safety Desk Assistant
Kerstin Hovestadt	Safety Desk Assistant
Linus Lauterbach	Safety Desk Assistant

S1.6 Members Of Quality Of Life Coordinating Centre

Gabriele Calaminus	TMG Member: Quality of Life Panel
Andreas Wiener	TMG Member: Quality of Life Panel
Katja Baust	Psychotherapist
Carmen Teske	Study Documentation
Karina Riemenschneider	Secretary

List S1.7: Expert Panels

Oncology Panel

The oncology panels of each group checked the protocol for content, consistency and accuracy on all topics surrounding study medication. Members of each group's oncology panel were appointed to represent the group in the TMG and the TSC. Some groups offered or required consultation with named members of the oncology panel on issues surrounding chemotherapy.

Pathology review panel

The purpose of the Pathology Review Panel was to ensure that the histopathological criteria for admission to the trial and histological response to chemotherapy were assessed in a timely and consistent fashion for all trial groups participating in EURAMOS 1. Each of the participating groups formed a pathology sub-panel. Together, these form the pathology panel of the EURAMOS 1 trial. Each of the 4 collaborating groups named one representative pathologist to represent their group in intergroup discussions. These four elected one pathologist to represent pathology in the Trial Management Committee.

Pathology review was to be undertaken for all patients within the trial. Both the diagnostic biopsy and the resection specimen histology were to be reviewed by a study pathologist. The process of pathology review is detailed in the protocol.

Biological Studies panel

Parallel biological studies were performed in selected groups for this trial lead by the biological studies panel. The biological studies panel were responsible for the development of protocols within their trial group and for ensuring the use of uniform methodologies between groups to allow data comparability.

Surgical panel

The surgical panel issued guidance surrounding tumour resection and reconstruction. Some groups offered or required consultation with members of the surgical panel prior to tumour surgery.

Radiology panel

The radiology panel issued guidance surrounding imaging of the primary tumour and of metastatic disease. Members of the panel assisted in determining metastatic status. Review of thorax CT scans was undertaken by members of the trial management group in conjunction with a panel radiologist. Central review of chest CT scans was requested for patients registered as having (possible) metastatic disease and fulfilling the following criteria: no lesion greater than 1 cm or less than 3 lesions of any size.

Radiotherapy panel

The radiotherapy panel issued guidance surrounding radiotherapy for insufficient margins or inoperable relapse. Some groups offered or required consultation with members of the radiotherapy panel prior to irradiating a specific patient.

Statistics panel

The statistics panel was involved in all aspects of trial design, data storage and management, and evaluation.

List S2: Independent Members Of Independent Trial Oversight Committees

(a) Independent Data Monitoring Committee

Name	Role
Barry Hancock	Chair: Sheffield, UK
Gerald Gilchrist	Member: Minnesota, USA
Otilia Dalesio	Member: Amsterdam, NL
Peter Høglund	Member: Lund, Sweden

(b) Trial Steering Committee

Name	Role
Stefano Ferrari	Chair: Bologna, Italy
Joseph Mirro	Member: Memphis, USA
Hans Strander	Member: Stockholm, Sweden
Robert Souhami	Member: London, UK
